# Insecticidal Activity of *Artemisia vulgaris* Essential Oil and Transcriptome Analysis of *Tribolium castaneum* in Response to Oil Exposure

**DOI:** 10.3389/fgene.2020.00589

**Published:** 2020-06-25

**Authors:** Shanshan Gao, Kunpeng Zhang, Luting Wei, Guanyun Wei, Wenfeng Xiong, Yaoyao Lu, Yonglei Zhang, Aoxiang Gao, Bin Li

**Affiliations:** ^1^Henan Joint International Research Laboratory of Veterinary Biologics Research and Application, Anyang Institute of Technology, Anyang, China; ^2^Jiangsu Key Laboratory for Biodiversity and Biotechnology, College of Life Sciences, Nanjing Normal University, Nanjing, China; ^3^College of Life Sciences, Nantong University, Nantong, China

**Keywords:** *Tribolium castaneum*, *Artemisia vulgaris*, insecticidal activity, development, reproduction, metabolism system

## Abstract

Red flour beetle (*Tribolium castaneum*) is one of the most destructive pests of stored cereals worldwide. The essential oil (EO) of *Artemisia vulgaris* (mugwort) is known to be a strong toxicant that inhibits the growth, development, and reproduction of *T. castaneum*. However, the molecular mechanisms underlying the toxic effects of *A. vulgaris* EO on *T. castaneum* remain unclear. Here, two detoxifying enzymes, carboxylesterase (CarEs) and cytochrome oxidase P450 (CYPs), were dramatically increased in red flour beetle larvae when they were exposed to *A. vulgaris* EO. Further, 758 genes were differentially expressed between EO treated and control samples. Based on Gene Ontology (GO) analysis, numerous differentially expressed genes (DEGs) were enriched for terms related to the regulation of biological processes, response to stimulus, and antigen processing and presentation. Our results indicated that *A. vulgaris* EO disturbed the antioxidant activity in larvae and partially inhibited serine protease (SP), cathepsin (CAT), and lipase signaling pathways, thus disrupting larval development and reproduction as well as down-regulating the stress response. Moreover, these DEGs showed that *A. vulgaris* indirectly affected the development and reproduction of beetles by inducing the expression of genes encoding copper-zinc-superoxide dismutase (CuZnSOD), heme peroxidase (HPX), antioxidant enzymes, and transcription factors. Moreover, the majority of DEGs were mapped to the drug metabolism pathway in the Kyoto Encyclopedia of Genes and Genomes (KEGG) database. Notably, the following genes were detected: 6 *odorant binding proteins* (*OBPs*), 5 *chemosensory proteins* (*CSPs*), 14 *CYPs*, 3 *esterases* (*ESTs*), 5 *glutathione S-transferases* (*GSTs*), 6 *UDP-glucuronosyltransferases* (*UGTs*), and 2 *multidrug resistance proteins* (*MRPs*), of which 8 *CYPs*, 2 *ESTs*, 2 *GSTs*, and 3 *UGTs* were up-regulated dramatically after exposure to *A. vulgaris* EO. The residual DEGs were significantly down-regulated in EO exposed larvae, implying that partial compensation of metabolism detoxification existed in treated beetles. Furthermore, *A. vulgaris* EO induced overexpression of *OBP*/*CYP*, and RNAi against these genes significantly increased mortality of larvae exposed to EO, providing further evidence for the involvement of *OBP*/*CYP* in EO metabolic detoxification in *T. castaneum*. Our results provide an overview of the transcriptomic changes in *T. castaneum* in response to *A. vulgaris* EO.

## Introduction

Insects are serious pests of stored products such as oilseeds, pulses, and cereals (Phillips and Throne, 2010; Wakil et al., 2010). These pests are distributed worldwide and cause huge economic losses (Flinn et al., 2010). The red flour beetle (*Tribolium castaneum*; Tenebrionidae) is one of the most economically crucial insect pests of stored-products (Salem et al., 2018) and a common pest of indoor food storage facilities (Wang Y. et al., 2015). The use of chemical fumigants (methyl bromide and phosphine) is currently one of the most effective methods for controlling stored-product insect pests (Athanassiou et al., 2015; Thompson and Reddy, 2016). Chemical insecticides, such as pyrethroids, phosphine, and dichlorvos, are also used to control these pests (Nayak et al., 2003; Hori and Kasaishi, 2005; Bomzan et al., 2018). However, improper and indiscriminate application of these insecticides can result in long-term undesirable effects on human health, non-targeted animals, and the environment (Dey, 2016). Because of the underlying ozone-depleting property of methyl bromide and the potential genotoxicity of phosphine to warm-blooded animals, the use of these insecticides has been restricted (Danse et al., 1984; Bell and Wilson, 1995; Kalsi and Palli, 2015). Therefore, the search for natural products of botanical-origin that have insecticidal activity has intensified in the scientific community (Isman, 2006).

Essential oils (EOs) of plants are mixtures of volatile compounds that have been widely used as bioactive agents. EOs are effective as insecticides (Qing et al., 2010), ovicides (Tunç et al., 2000), antifeedants (Huang et al., 2000), oviposition inhibitors (Ho et al., 1996), and repellents (Ogendo et al., 2008). Moreover, these chemicals affect certain biological parameters such as the life span, growth rate, and reproduction of insects (Naseri et al., 2017). The EO of *Ocimum basilicum* (Labiatae) exhibits highly toxic and repellent activity against *T. castaneum* adults when applied topically or impregnated on filter paper, grains, or glass pebbles (Obeng-Ofori and Reichmuth, 1997). Similar activities have been pointed out for EOs from *Artemisia annua* (Asteraceae) and *Baccharis salicifolia* (Asteraceae) (García et al., 2005; Goel et al., 2007), *Trachyspermum ammi* (Umbelliferae), *Nigella sativa* (Ranunculaceae), and *Anethum graveolens* (Umbelliferae) (Jana and Shekhawat, 2010; Khani and Asghari, 2012; Soni et al., 2016).

Numerous studies have reported the insecticidal proprieties of EOs made from *Artemisia* species against a wide range of insect pests. *Artemisia* is a genus of fragrant annual herb species belonging to the Compositae family, with a wide distribution in Asia, Europe, and North America (Kordali et al., 2006; Jiang et al., 2019). Previous studies show that *Artemisia* EOs exhibit antifeedant, repellent, and insecticidal activities against various insect pests including *T. castaneum*, *Callosobruchus maculatuss* (Bruchidae), *Rhyzopertha dominica* (Bostrichidae), and *Plodia interpunctella* (Phycitinae) (Tripathi et al., 2000; Sharifian et al., 2012; Borzoui et al., 2016). Furthermore, *Artemisia* EOs and their constituents also exhibit sublethal effects on the survival, fecundity, development, and life table parameters of *Sitophilus granaries* (Curculionidae), *Tribolium confusum* (Tenebrionidae), *C. maculatuss*, *P. interpunctella*, *Trogoderma granarium* (Dermestidae), and *T. castaneum* (Kordali et al., 2006; Wang et al., 2006; Stamopoulos et al., 2007; Izakmehri et al., 2013; Borzoui et al., 2016; Nouri-Ganbalani and Borzoui, 2017). The repellent and fumigant activities of *Artemisia vulgaris* (Asteraceae) EO has been demonstrated against *Musca domestica* (Muscidae) and the stored-product insect pest *T. castaneum* (Wang et al., 2006; Alizadeh et al., 2012). Furthermore, the insecticidal activity of *A. vulgaris* EO has been reported against *C. maculatus*, *R. dominica*, and *T. castaneum* (Sharifian et al., 2013). The insecticidal and larvicidal properties of *A. vulgaris* EO have been attributed to the presence of camphene, a chloro derivative of camphene, and α-Thujone (Pandey and Singh, 2017). Camphor has also been reported as one of the active components of *A. vulgaris* EO, which possesses moth repellent properties, and therefore has been used as a preservative in pharmaceuticals and cosmetics (Corrêa-Ferreira et al., 2014).

Currently, three modes of action of plant EOs have been identified against insect pests: (1) action on the nervous system of insects, thus suppressing normal growth, development, metamorphosis, and reproduction; (2) suppression of mitochondrial membrane respiratory enzymes; (3) regulation of oxygen consumption and carbon dioxide released (Pare and Tumlinson, 1999; Kostyukovsky et al., 2002; Mansour and Abdel-Hamid, 2015; Nascimento et al., 2015; Oboh et al., 2017). Insect genomes encode a variety of detoxification enzymes, including carboxylesterase (CarE, also called CCE/EST/CES), cytochrome oxidase P450 (CYP), and glutathione *S*-transferase (GST), to cope with xenobiotic compounds (Li et al., 2013; Zhang et al., 2013; Xiong et al., 2019b). Determination of the activity of these metabolic enzymes in insects after insecticide application has been widely used to try to understand the insecticidal mechanism of xenobiotic compounds (Francis et al., 2005). On the other hand, transcriptional regulation of gene expression in insects plays an significant role in insect response to various stresses (Chen et al., 2016).

However, molecular mechanisms underlying the insecticidal effects of *A. vulgaris* EO on stored-product insect pests remains unclear. In this paper, we aimed to assess the lethal effects of *A. vulgaris* EO against *T. castaneum* under laboratory conditions. Furthermore, we examined roles of the three most important detoxification enzymes (CarEs, CYPs, and GSTs) in the response of *T. castaneum* to *A. vulgaris* EO. To further explore how *A. vulgaris* affects the physiological activity of *T. castaneum*, we performed comparative transcriptome analyses of *T. castaneum* larvae treated with or without *A. vulgaris* EO. To validate the sequencing results and survey expression variation of response time-course for key responding genes, we provided bioassay analysis for a group of differentially expressed genes (DEGs) selected randomly and key responding genes. This paper provides the first overview of the molecular events underlying the response of *T. castaneum* to *A. vulgaris* EO. Our results provide a strong foundation for the development of plant EOs as novel, natural, and environmentally friendly insecticides.

## Materials and Methods

### Insect Culturing, Extraction, and Insecticidal Efficacy Assay of *A. vulgaris* EO

The *T. castaneum* Georgia-1 (GA-1) strain was used in this study. Insects were reared in whole wheat flour containing 5% brewer’s yeast in a growth chamber maintained at 30°C under a 14-h light/10-h dark cycle as described previously (Xiong et al., 2019b).

*Artemisia vulgaris* EO was extracted using the subcritical butane extraction apparatus (Henan Subcritical Biological Technology Co., Ltd., Anyang, China) with the following parameters: liquid:solid ratio = 30:1, temperature = 45°C, extraction time = 34 min, and particle size = 0.26 mm. The extract was subjected to hydrodistillation in a Clevenger-type apparatus for 2 h. To separate the EO from residual water, the oil/water emulsion was collected and stored at 4°C overnight. The EO was then transferred and stored in a glass bottle at room temperature until further use (Jiang et al., 2019).

To test the insecticidal efficacy, *A. vulgaris* EO was dissolved in acetone at seven different concentrations (1, 1.25, 1.7, 2.5, 5, 10, and 100%). Twenty-day-old *T. castaneum* larvae were used to determine the contact activity of *A. vulgaris* EO, as described by Lu et al. (2012). Fifteen larvae were treated with approximately 50 μL of each EO solution or acetone (control) for 1 min and then placed on Whatman filter paper for air drying for approximately 2 min. Subsequently, the larvae were transferred into an 8-ml glass vial and maintained under standard conditions. Mortality was recorded at 24, 48, and 72 h after treatment. Larvae were considered dead if they were unable to move or show response when disturbed with a pair of tweezers or a brush. Each experiment was repeated at least three times.

### Assessment of Enzyme Activities

During enzyme preparation, we treated another set of test insects with *A. vulgaris* EO at 5% concentration (approximately median lethal concentration, LC50) or acetone (control); larvae were sampled at 12, 24, 48, 60, and 72 h, when they were weighed and washed twice or three times with pre-cooled saline. Then, larvae were dried using a piece of filter paper and ground in liquid nitrogen using a mortar and pestle. The resultant powder was transferred to a centrifuge tube to obtain 10% tissue homogenate in physiological saline and the issue suspension was centrifuged at 3,500 rpm for 10 min at 4°C. The supernatant was taken into a new tube for the enzyme activity test. Enzyme activity estimation and calculation method were refereed as previously described (Guo et al., 2012; Xiong et al., 2019b).

CarE enzyme activity was quantified using a spectrophotometric assay kit (catalog no. A133-1-1; Nanjing Jiancheng Bioengineering Institute, Nanjing, China), according to the manufacturer’s protocol, from proteins in the tissue samples that were extracted using a protein extraction kit (catalog no. A045-4-2; Nanjing Jiancheng Bioengineering Institute, Nanjing, China). The extracted protein samples were incubated with 1-naphthalenol, and following incubation at room temperature for 30 min with agitation, absorbance was measured at 450 nm.

Total CYP activity was measured using a linked immunosorbent assay (ELISA) method using a commercially available kit (catalog no. H190-1, Nanjing Jiancheng Bioengineering Institute, Nanjing, China), by adding 50 μL of standards (7.5, 15, 30, 60, and 120 ng/mL) to the wells and drawing an A 4-parameter calibration curve (CurveExpert 1.4) for protein quantification. Then, 50 μL of the sample to be tested was added to the sample wells, with blank standards as a control, and washed, to which horseradish peroxidase (HRP) labeled antibody was also added. Samples were washed again, and HRP substrate was added and reacted for 5 min in the dark at 37°C, before absorbance at 450 nm was recorded.

Glutathione *S*-transferase activity test was conducted using a GST detection kit (catalog no. A004-1-1, Nanjing Jiancheng Bioengineering Institute, Nanjing, China), where one unit of activity was defined as a 1 μmol decrease in glutathione concentration in 1 mg of tissue protein for 1 min at 37°C, when the effects of non-enzymatic reactions are eliminated; absorbance was measured at 412 nm. Total protein concentration was determined using a BCA protein assay kit (catalog no. A045-4-2; Nanjing Jiancheng Bioengineering Institute, Nanjing, China), according to the manufacturer’s instructions.

### RNA Isolation, cDNA Synthesis, and RNA-Seq

Because preliminary biochemical assays showed that CarE and CYP enzyme activities were significantly enhanced in larvae treated with 5% EO at 36 h, six samples (including three control and three EO treated samples harvested at the 36-h time point; each sample contained six larvae) were selected for RNA-Seq analysis. Total RNA was isolated from *T. castaneum* larvae using RNAiso^TM^ Plus (TaKaRa, Kyoto, Japan), according to the manufacturer’s instructions, and treated with DNaseI at 37°C for 20 min to remove residual DNA contamination. The absorbance ratio at 260 and 280 nm (*A*_260_/*A*_280_) of total RNA was measured to assess the yield and purity of the sample, and the integrity of total RNA was assessed by electrophoresis on 1.5% agarose gel using an Agilent 2100 Bioanalyzer. Intact total RNA samples (1 μg) with an *A*_260_/*A*_280_ ratio > 1.8 were used for mRNA purification with Oligo(dT) magnetic beads. Then, the purified mRNA was fragmented and reverse transcribed to synthesize first-strand cDNA using Moloney murine leukemia virus reverse transcriptase and Oligo(dT)18 primer (TaKaRa, Kyoto, Japan). Double-stranded cDNA (dscDNA) was generated using the N6 random primer. The dscDNAs were end-repaired by the addition of phosphate at the 5′ end and nucleotide A at the 3′ end, and then ligated to an adaptor at the 3′ end. Two sequence-specific primers were used to amplify the ligation products. The PCR products were heat-denatured and circularized using splint oligo and DNA ligase. The resulting products were sequenced at Beijing Genomics Institute (BGI; Shenzhen, China) on the BGISEQ-500 platform^1^. Raw sequence reads were deposited in the NCBI Sequence Read Archive (SRA) database under the following accession numbers: control: SRR7646221, SRR7646223, and SRR7646224; EO treatment: SRR7646222, SRR7646225, and SRR7646226.

### Filtering and Mapping of RNA-Seq Data

Raw sequence reads were filtered using FASTX^2^ to remove adaptor sequences, reads with >10% unknown nucleotides, low-quality reads (percentage of low-quality nucleotides >50% or nucleotides with sequence quality ≤5), and short reads (<20 bp). The *T. castaneum* genome sequence and corresponding annotations were downloaded from the BeetleBase database^3^ (version: *T. castaneum* 5.2), and clean reads were mapped to the reference gene sequences using Bowtie2 (Stephen et al., 2008) and to the *T. castaneum* reference genome using hierarchical indexing for spliced alignment of transcripts (HISAT) (Daehwan et al., 2015).

### Determination of Gene Expression and Identification of DEGs

Gene expression levels were quantified using the RNA-Seq of the Expectation Maximization (RSEM) software package (Bo and Dewey, 2011). The relative transcript level of each gene was normalized and evaluated using fragments per kilobase of exons per million fragments mapped (FPKM) values. Repeatability among the three biological replicates was assessed using cluster analysis calculated by the Euclidean method. The mean FPKM value of three biological replicates of each gene was calculated to determine the expression level of each gene, and then genes with FPKM values ≥ 0.5 were analyzed further. The DEGs identified between the control and EO treatment groups were screened using the NOISeq package^4^ (Tarazona et al., 2011) of R. DEGs with a fold-change (FC) ≥ 2(| log2 ratio| ≥ 1 and divergence probability ≥ 0.8 were selected for further analysis.

### Functional Annotations of DEGs

To understand the main biological and molecular functions of DEGs, annotations were performed using the Gene Ontology (GO) database^5^, according to the annotation information in the non-redundant (NR) database, and categorized into three standardized gene functional classifications (molecular functions, cellular components, and biological processes) using the Blast2GO pipeline (Ana et al., 2005). The Kyoto Encyclopedia of Genes and Genomes (KEGG^6^) database is the main public repository of known pathways. KEGG annotations were performed using the NCBI BLAST tool for all *T. castaneum* genes, followed by KEGG enrichment analysis of DEGs using the KOBAS 2.0 software^7^. Significance levels of all KEGG and GO terms were corrected by controlling the false discovery rate (FDR) of multiple pairwise comparisons, and terms with significantly enriched values (FDR < 0.05) were determined.

### Validation of RNA-Seq Data by Quantitative Real-Time PCR (qRT-PCR)

To validate RNA-Seq results, the expression of 16 randomly selected DEGs was analyzed by qRT-PCR using the same RNA samples as those used for RNA-Seq analysis. The qRT-PCR was performed on a StepOnePlus Real-Time PCR System (Applied Biosystems, Foster City, CA, United States) using SYBR Green Master Mix (Roche, Indianapolis, IN, United States) as a fluorescent dye, according to the manufacturer’s instructions. Three biological replications were performed for each experiment. Relative transcript levels of genes were determined using *T. castaneum ribosomal protein S3* (*rps3*; GenBank accession number CB335975) as the internal reference gene, and gene expression levels were calculated using the 2^–ΔΔCT^ method (Livak and Schmittgen, 2001). Primers used for qRT-PCR are listed in Supplementary Table S1.

### Induction of *TcCYP4BN6* and *TcOBPC11* Induction in Response to the *A. vulgaris* EO

Two genes (*TcCYP4BN6* and *TcOBPC11*) that were significantly induced by *A. vulgaris* EO were selected for further study. To measure *TcCYP4BN6* and *TcOBPC11* induction patterns after EO treatments, 20-day-old larvae were first collected and then separated into three groups. Briefly, approximately 36 synchronous individuals in each group were loaded into 1.5-mL EP tubes and exposed to 120 μL of 5% *A. vulgaris* EO or acetone (3 beetles/10 μL). After soaking for 1 min, the treated larvae in each group were placed on filter paper and allowed to air dry for 2 min. Each group was then transferred to an 8-mL glass vial and maintained under standard conditions as previously described (Wei et al., 2019). The acetone exposure group served as the negative control group in this study.

### RNAi to Evaluate the Roles of *TcCYP4BN6* and *TcOBPC11* Response to the *A. vulgaris* EO

To further investigate the functions of *TcCYP4BN6* and *TcOBPC11* in response to the *A. vulgaris* EO, RNA interference (RNAi) was used. Here, primers tailed with T7 promoters on the 5′ side were used to synthesize isoform-specific double stranded RNAs (dsRNAs), which target specific region for each isoform (Supplementary Table S1). dsRNAs are synthesized as previously described (Bi et al., 2019). A total of 200 ng of dsRNAs were injected into each larva of *T. castaneum*. Further, the collected 20-day-old larvae were separated into three groups and then used for ds-*TcCYP4BN6* injection, ds-*TcOBPC11* injection, and ds-*GFP* injection. The insects were further treated with 5% *A. vulgaris* EO to evaluate mortality. Prior to *A. vulgaris* EO exposure, RNAi efficiency was assessed by determining the transcript levels of the target genes after RNAi application. Further, at fifth day following dsRNA injection, three larvae were randomly selected for RNA extraction, and the RNA samples were employed in qRT-PCR. Briefly, approximately 50 μL of *A. vulgaris* EO was applied to the 15 ds-*TcCYP4BN6* injection, ds-*TcOBPC11* injection, and ds-*GFP* injection larvae. The mortalities of the larvae in the different treatment groups were recorded from 24 to 72 h after EO exposure. In this assay, the beetles were considered dead if they were unable to move and failed to respond when disturbed with a tweezer or brush. Each bioassay was replicated three times.

### Statistical Analysis

Mortality data from the insecticidal efficacy assay of *A. vulgaris* EO were analyzed for the LC50 values and their 95% confidence intervals (95% CIs) by probit analysis using the SPSS program (SPSS Inc., Chicago, IL, United States). The effect of essential oils on enzymatic activities, the gene expression data, the mean values of the RNAi-treated versus the mean values of the control insects were compared by Student’s *t*-test and one-way analysis of variance (ANOVA) in combination with a Fisher’s least significant difference (LSD) multiple comparison tests, respectively, by using the SPSS statistics program (Chicago, IL, United States). All data are presented as the mean ± standard error (SE). Differences were considered significant at *P*-value < 0.05.

## Results

### Insecticidal Activity of *A. vulgaris* EO

**FIGURE 1:**
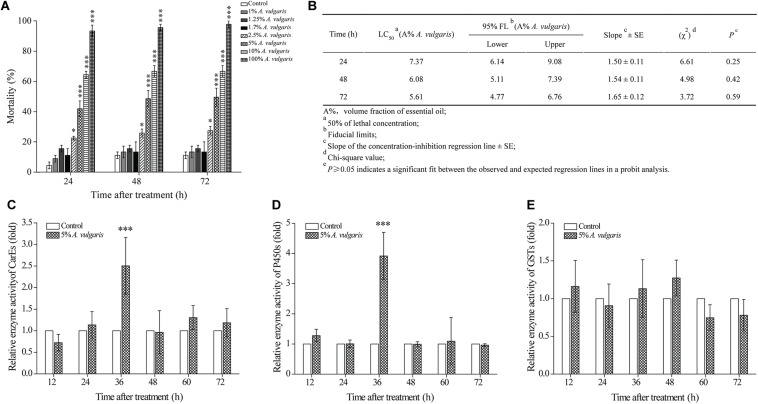
**(A)** The effect of *Artemisia vulgaris* essential oil (EO) on *Tribolium castaneum* larvae. Larvae were treated with eight different concentrations (0.1, 1, 1.25, 1.7, 2.5, 5, 10, and 100%) of *A. vulgaris* EO or with acetone (control). Larval mortality was observed at 24, 48, and 72 h post-treatment. **(B)** Contact toxicity of *A. vulgaris* EO against *T. castaneum* larvae. At different times with 5% *A. vulgaris* EO on carboxylesterase (CarEs) **(C)**, cytochrome P450 (CYPs) **(D)**, and glutathione S-transferase (GSTs) **(E)** in larval *T. castaneum in vivo*. Fold change of the total enzyme activity was calculated by dividing the enzyme activity of each treatment by that of the acetone only control, which had been ascribed an arbitrary value of one. The times of 12, 24, 36, 48, 60, and 72 h were six different test points after insecticide treatment. Data represent mean ± SE of three independent experiments. Asterisks indicate significant differences between the control and EO treatment groups (^∗^*P* < 0.05, ^∗∗∗^*P* < 0.001; Student’s *t*-test).

To investigate the insecticidal activity of *A. vulgaris* EO against *T. castaneum* larvae, we performed contact assays. Larvae treated with 1–1.7% *A. vulgaris* EO showed a slight increase in the mortality rate at 48, 60, and 72 h. By contrast, in larvae treated with EO concentrations of 2.5, 5, 10, and 100%, the cumulative mortality reached 27.41% ± 2.82%, 49.52% ± 5.97%, 66.67% ± 3.85%, and 99.78% ± 2.22%, respectively, at 72 h (Figure 1A). At the same dose, slightly increased effect of exposure was observed over the time course of 24–48–72 h. The largest concentration of essential oils (100% *A. vulgaris* EO) caused the mortality of 93.33% ± 3.84%, 95.56% ± 2.22%, and 97.78% ± 2.22% in *T. castaneum* after 24, 48, and 72 h of EO treatment, respectively. The corresponding LC50 values were 6.14, 5.11, and 4.77% *A. vulgaris* EO, respectively (Figure 1B). These results demonstrate that the EO caused dose-dependent increase in larval mortality, while there were not apparent time-dependent effects.

### Effect of *A. vulgaris* EO on Enzyme Activities in *T. castaneum*

Next, we determined the effect of *A. vulgaris* EO on the activities of CarE, CYP, and GST enzymes in *T. castaneum* larvae. In larvae treated with 5% EO (approximately LC50 for 72 h), activities of CarE and CYP enzymes were increased in a similar pattern from 12 to 72 h (Figures 1C,D). Activities of both CarE and CYP enzymes in EO treated larvae reached a peak at 36 h (2.50- and 3.92-fold, respectively) and declined thereafter (Figures 1C,D). By contrast, GST activity in EO treated larvae showed no significant change from 12 to 72 h (Figure 1E).

### RNA-Seq and Read Alignment

To explore the gene expression profiles of *T. castaneum* in response to *A. vulgaris* EO treatment, six RNA-Seq libraries (three control and three EO treatment groups) were sequenced. An average of 23,647,023 raw reads were generated, from which 23,271,263 clean reads were obtained after filtering out low-quality sequences (Table 1). The ratio of clean reads to raw reads for the six libraries ranged from 99.66 to 99.85%. On average, 53.578% of the control reads mapped either to a unique location (52.16%) or to multiple locations (1.61%) in the *T. castaneum* genome. A similar proportion (54.74%) of the EO treatment group reads mapped to the *T. castaneum* genome, most of which mapped to a unique location (53.34%), and a small proportion mapped to multiple locations (1.40%; Table 1). These data demonstrate that all six libraries were of high quality (Kim et al., 2016).

**TABLE 1:** Summary of read numbers based on the RNA-sequencing data.

Category	Control			Treatment		
	Control_rep1	Control_rep2	Control_rep3	5% *A. vulgaris*_rep1	5% *A. vulgaris*_rep2	5% *A. vulgaris*_rep3
Total reads number	23203638	23649862	24087570	24054117	24071256	21688417
Total mapped reads	13245867	10272901	14644323	15017197	10780866	12359145
	(57.09%)	(43.44%)	(60.80%)	(62.43%)	(44.79%)	(56.99%)
Perfect match	10762958	8295964	11945631	12317982	8834002	10034020
	(46.38%)	(35.08%)	(49.59%)	(51.21%)	(36.70%)	(46.26%)
Mismatch	2482909	1976937	2698692	2699215	1946864	2325125
	(10.70%)	(8.36%)	(11.20%)	(11.22%)	(8.09%)	(10.72%)
Unique match	12892239	9909736	14218684	14604563	10479759	12094489
	(55.56%)	(41.90%)	(59.03%)	(60.72%)	(43.54%)	(55.76%)
Multi-position match	353628	363165	425639	412634	301107	264656
	(1.52%)	(1.54%)	(1.77%)	(1.72%)	(1.25%)	(1.22%)
Total unmapped reads	9957771	13376961	9443247	9036920	13290390	9329272
	(42.91%)	(56.56%)	(39.20%)	(37.57%)	(55.21%)	(43.01%)

An average of 12,156 and 12,239 genes (80 to ≥2,000 bp in length) were detected in the control and EO treatment samples, respectively. Based on their length, these genes were divided into five categories. Approximately 50% of the genes were 500–1,500 bp in length, 20.19% ranged from 500 to 1,000 bp, and 22.47% ranged from 1,000 to 1,500 bp (Supplementary Figure S1A). The distribution of genes in each category was similar between the control and treatment samples (Supplementary Figure S1B). The majority of genes showed FPKM values ranging from 10 to 100 in both control (6,424 genes, 52.84%) and EO treatment groups (6,382 genes, 52.15%; Supplementary Figure S1B). A few genes showed FPKM values >1,000, indicating that only a small proportion of genes were expressed to high levels. No significant differences in FPKM values were detected between the control and EO treatment groups (Supplementary Figure S1B).

Next, we performed sequence saturation analysis to determine whether the number of detected genes increased proportionally with the number of sequence reads. The results indicated that the number of detected genes stopped increasing beyond 1.5 million sequence reads (Supplementary Figures S2G–L). To determine mRNA expression, redundancy and heterogeneity were considered as two key characteristics. Further, a small proportion of mRNAs were expressed at high levels, while the majority was expressed at low levels. Thus, we used the distribution of tag expression to assess the normality of RNA-seq data. In addition, the distribution of distinct tags over different tag abundance categories showed that the proportions of the control and EO treatment libraries were nearly equal (Supplementary Figures S2G–L). Both types of libraries exhibited similar distribution patterns, with more than 50% of detected genes showing a coverage of >80% (Supplementary Figures S2A–F). In total, the control and EO treatment libraries yielded 12,684 genes, thus providing abundant data for analyzing the influence of *A. vulgaris* EO on the physiological characteristics of *T. castaneum* larvae.

### GO and KEGG Enrichment Analyses of DEGs

**FIGURE 2:**
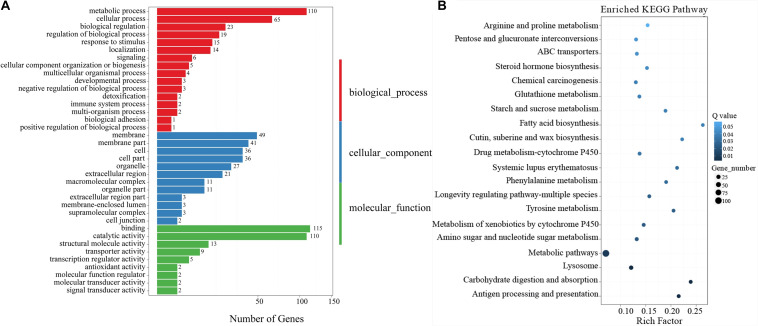
**(A)** Gene Ontology (GO) enriched terms of differentially expressed genes (DEGs) of *T. castaneum* after exposure of its larvae to 5% *A. vulgaris* essential oil (EO) versus the control group. The *x*-axis is the number of DEGs involved in each term. The *y*-axis lists the sub-GO terms under categories of biological processes, cellular components, and molecular functions. **(B)** The most enriched Kyoto Encyclopedia of Genes and Genomes (KEGG) pathways of *T. castaneum* after exposure of its larvae to 5% *A. vulgaris* EO. Pathway significance is shown together with *Q*-value (color), rich factor (vertical ordinate), and a number of involved genes (size of circles).

Functional annotations of DEGs were performed by GO enrichment analysis. A total of 142, 99, and 198 DEGs (*P* ≤ 0.05) were assigned to the cellular component, molecular function, and biological process categories, respectively, and further divided into 37 sub-categories (16 in biological processes, 12 in cellular components, and 9 in molecular function categories; Figure 2A). In these categories, regulation of biological processes (GO:0050789), developmental processes (GO: 0032502), and response to stimulus (GO: 0050896) were closely associated with the influence of *A. vulgaris* EO on *T. castaneum* larvae. A total of 19 DEGs encoding Wnt 8a, netrin receptor UNC5C, and myosin 9, which play significant roles in insect eclosion and reproduction, were associated with the regulation of biological processes (GO:0050789; Table 2). The GO term response to stimulus (GO: 0050896) contained 15 DEGs, including *Peroxidase*, *Defensin1*, and *Defensin2* (Supplementary Table S4), which were involved in immunity and stress responses simultaneously. Moreover, the GO terms binding (GO: 0005488), catalytic activity (GO: 0003824) with 109 DEGs, and metabolic processes (GO: 0008152) contained 115, 109, and 100 DEGs, respectively (Figure 2A).

**TABLE 2:** Differentially expressed genes (DEGs) involved in regulation of biological process.

Gene ID	Log_2_ ratio (T/C)	Regulation (T/C)	Protein	*P*-value	Phenotype after RNAi of DEGs in *T. castaneum*^a^
LOC664422	2.50	Up	Gadd45	0	/
LOC663364	2.10	Up	DETS 6	5.37E-218	/
LOC662363	2.05	Up	ADAMTS7	0	/
LOC100141722	1.58	Up	Octopamine receptor 1	7.54E-05	/
LOC103314939	1.37	Up	Transcription factor GATA 4	7.94E-35	/
LOC663803	1.10	Up	Eukaryotic translation initiation factor 4E-binding protein 2	3.44E-205	/
LOC660084	−1.00	Down	Wnt 8a	7.50E-06	/
LOC664175	−1.02	Down	Netrin receptor UNC5C	5.56E-156	/
LOC663899	−1.12	Down	Myosin 9	0	/
LOC658656	−1.13	Down	Apterous	9.30E-31	/
LOC656430	−1.15	Down	Fork head domain-containing protein FD4	4.07E-19	/
LOC100142549	−1.17	Down	Targeting protein for xklp2	9.34E-48	80% embryo/egg not developed/not fertilized^c^
LOC641604	−1.22	Down	Orthodenticle 2	2.50E-05	/
LOC656829	−1.27	Down	Transmembrane protein 80	6.07E-10	/
LOC641600	−1.29	Down	Scr	2.78E-87	30%^b^; 30 ∼ 50% embryo/egg with embryonic tissue, no cuticle^c^
LOC662160	−1.31	Down	TIMELESS-interacting protein	9.24E-22	/
LOC662392	−1.47	Down	Histone H2A	0	/
LOC103314024	−1.81	Down	Enhancer of split malpha protein	5.57E-43	/
LOC659737	−2.04	Down	Neuropeptide Y receptor	5.35E-05	/

*C, control; T, 5% A. vulgaris treatment; Gadd45, growth arrest and DNA damage-indueible genes; DETS 6, DNA-binding protein DETS-6; ADAMTS7, A disintegrin and metalloproteinase with thrombospondin motifs 7; Scr, Sex combs reduced; ^a^metamorphosis and survival after RNAi were analyzed (http://ibeetle-base.uni-goettingen.de); ^b^Lethalities 11 days after larval injection (includes death as larva, prepupa, and pupa); ^c^14 days after female pupal injection.*

The results of KEGG analysis showed that 511 DEGs were assigned to 262 biological pathways (Supplementary Table S2). A total of 19 pathways including antigen processing and presentation (ko04612), carbohydrate digestion and absorption (ko04973), lysosome (ko04142), and metabolism of xenobiotics by cytochrome P450 (ko00980) were significantly enriched in DEGs compared with the whole transcriptome background, with a *Q*-value ≤ 0.05 (Figure 2B and Supplementary Table S2). A total of 28 DEGs were involved in the lysosome pathway (ko04142) including *lysosomal alpha-mannosidase like*, *beta-glucuronidase* (*BG*), *cathepsin L precursor* (*CatL precursor*), *alpha-N-acetylgalactosaminidase*, *cathepsin B1* (*CatB1*), *arylsulfatase B-like*, *serine protease P38* (*SPP38*), *MD-2-related lipid-recognition* (*ML*), *Lipase 1*, *Lipase 3-like*, and *Lipase 4* (Table 3), which probably contributed to the development and reproduction of *T. castaneum*. Moreover, 14 DEGs participated in antigen processing and presentation (Table 3 and Supplementary Table S5), of which six included members of the lipase family that overlapped with the lysosome pathway. These six genes are likely involved in the stress response and innate immunity as well as in the reproduction and development of larvae. Notably, 44 DEGs were responsible for three drug metabolism pathways (ko00980, ko00982, and ko00983), which contained genes encoding flavin-containing monooxygenase (FMO), esterase (EST), UDP-glucuronosyltransferase (Ugt), BG, xanthine dehydrogenase (XDH), GST, and CYP enzymes (Table 4). The DEGs also encoded odorant binding proteins (OBPs) and chemosensory proteins (CSPs), which have been suggested to be involved in drug metabolism in *Plutella xylostella* (Bakhetia et al., 2005). Together, our results demonstrate considerable differences in physiological processes between EO treatment and control samples.

**TABLE 3:** Differentially expressed genes associated with the lysosome pathway.

Gene ID	Log_2_ ratio (T/C)	Regulation (T/C)	*P*-value	Protein	Phenotype after RNAi of DEGs in *T. castaneum*^a^
LOC658343	2.92	Up	1.06E-24	CP 1	/
LOC660669	2.62	Up	8.30E-75	Cat L precursor	/
LOC659367	2.08	Up	1.84E-77	Cat L precursor	/
LOC664569	2.04	Up	0	Beta-hexosaminidase subunit alpha	/
LOC663215	1.96	Up	3.22E-17	MFS15	/
LOC657117	1.81	Up	1.26E-06	Cat B1 like	/
LOC107398158	1.79	Up	7.41E-05	Cat L1 like	/
LOC100141821	1.48	Up	4.30E-12	Snmp1	/
LOC659039	1.41	Up	3.36E-28	Lipase member K	/
LOC103312286	1.30	Up	5.53E-295	Lipopolysaccharide	/
LOC664564	1.28	Up	3.89E-07	Glucosylceramidase like	/
LOC660551	1.26	Up	1.04E-307	Cat L	/
LOC103313498	1.10	Up	0	Crammer	/
LOC103314303	1.03	Up	1.29E-19	CD63 antigen like	/
LOC661588	1.03	Up	4.25E-67	Arylsulfatase B	/
LOC656698	−1.11	Down	2.40E-18	Lysosomal alpha-mannosidase like	/
LOC657825	−1.12	Down	1.27E-15	BG	/
LOC660368	−1.16	Down	3.69E-175	Cat L precursor	100%^b^, 40% vitellogenic egg chamber not present^c^
LOC662197	−1.28	Down	9.74E-90	Alpha-*N*-acetylgalactosaminidase	/
LOC663117	−1.30	Down	7.73E-28	Cat B1	/
LOC659525	−1.32	Down	5.41E-84	Arylsulfatase B like	/
LOC655752	−1.36	Down	9.57E-12	Serine protease P38	/
LOC103312725	−1.63	Down	3.19E-11	ML	/
LOC661631	−1.75	Down	8.99E-81	Lipase 1	/
LOC659587	−1.78	Down	0	Arylsulfatase B like	/
LOC659121	−1.82	Down	2.14E-132	Lipase 1 like	100% previtellogenic egg chamber orientation irregular^c^
LOC661718	−2.14	Down	6.36E-24	Lipase 3 like	/
LOC661966	−2.97	Down	2.16E-296	Lipase 4	20%^b^

*C, control; T, 5% A. vulgaris treatment; CP, cysteine proteinase; Cat, cathepsin; Snmp1, sensory neuron membrane protein 1; MFS15, major facilitator superfamily transporter 15; BG, beta-glucuronidase; ML, MD-2-related lipid-recognition; ^a^metamorphosis and survival after RNAi were analyzed (http://ibeetle-base.uni-goettingen.de); ^b^Lethalities 11 days after larval injection (includes death as larva, prepupa, pupa); ^c^13 days after female pupal injection.*

**TABLE 4:** Differentially expressed genes (DEGs) involved in drug metabolism.

Gene ID	Log_2_ ratio (T/C)	Regulation (T/C)	*P*-value	Protein	Metabolism detoxification process
LOC656161	4.28	Up	2.26E-65	OBPC11	Phase 0
LOC660270	4.03	Up	0	CYP4BN6	Phase I
LOC100240681	2.12	Up	6.93E-09	OBPC17	Phase 0
LOC664471	2.04	Up	5.36E-52	CYP6BQ7	Phase I
LOC658048	2.03	Up	2.33E-37	CYP6A2	Phase I
LOC107398166	1.91	Up	1.98E-07	Ugt86Dg	Phase II
LOC664595	1.91	Up	2.23E-217	OBP10	Phase 0
LOC662506	1.87	Up	0	α-EST5	Phase I
LOC657602	1.76	Up	3.47E-76	GSTs6	Phase II
LOC103313315	1.63	Up	6.44E-23	CYP351A2	Phase I
LOC100141953	1.58	Up	2.82E-06	Ugt2B7-like	Phase II
LOC656937	1.47	Up	1.65E-114	MRP4	Phase III
LOC661102	1.43	Up	1.12E-09	α-EST2	Phase I
LOC664599	1.41	Up	5.71E-155	OBPC02	Phase 0
LOC656243	1.40	Up	0	OBPC12	Phase 0
LOC661621	1.37	Up	0	α-EST6	Phase I
LOC661270	1.36	Up	2.72E-31	CSP12	Phase 0
LOC657697	1.33	Up	0	CSP20	Phase 0
LOC664285	1.31	Up	2.63E-37	CYP9Z2	Phase I
LOC657454	1.27	Up	2.98E-30	CYP9AC1	Phase I
LOC661469	1.24	Up	0.000982	CSP17	Phase 0
LOC656120	1.22	Up	1.19E-27	Ugt2B16	Phase II
LOC659847	1.15	Up	4.96E-63	MRP49	Phase III
LOC659009	1.13	Up	4.50E-45	GSTs7	Phase II
LOC656770	1.12	Up	6.93E-55	CYP6BK11	Phase I
LOC661219	1.12	Up	1.22E-37	CSP11	Phase 0
LOC657560	1.07	Up	3.78E-13	CYP6A14	Phase I
LOC657825	−1.12	Down	1.27E-15	BG	Phase II
LOC100142486	−1.17	Down	1.98E-46	Ugt86Dc	Phase II
LOC656306	−1.22	Down	3.25E-57	CYP4BN5	Phase I
LOC655331	−1.24	Down	5.47E-242	GSTe7	Phase II
LOC655415	−1.33	Down	3.03E-92	GSTe8	Phase II
LOC658886	−1.42	Down	1.80E-08	XDH	Phase I
LOC661967	−1.48	Down	7.35E-79	Ugt2B20	Phase II
LOC659878	−1.51	Down	1.31E-71	CYP4Q1	Phase I
LOC662300	−1.99	Down	3.03E-22	CYP351A3	Phase I
LOC661799	−2.15	Down	9.46E-10	CSP8	Phase 0
LOC103313826	−2.22	Down	8.77E-05	FMO 2	Phase I
LOC664598	−2.25	Down	0	OBPC01	Phase 0
LOC660846	−2.44	Down	7.10E-103	Ugt2C1	Phase II
LOC662337	−2.49	Down	6.97E-127	CYP4C1	Phase I
LOC107398495	−2.77	Down	7.86E-25	GSTe4 like	Phase II
LOC664475	−2.87	Down	5.91E-05	CYP6A20	Phase I
LOC661930	−3.09	Down	5.08E-05	CYP349A1	Phase I

*C, control; T, 5% A. vulgaris treatment; OBP, odorant binding protein; CYP, cytochrome P450; Ugt, UDP-glucuronosyltransferase; EST, esterase; GST, glutathione S-transferase; MRP, multidrug resistance protein; BG, beta-glucuronidase; XDH, xanthine dehydrogenase; FMO, flavin-containing monooxygenase.*

### Expression Profiles of DEGs

**FIGURE 3:**
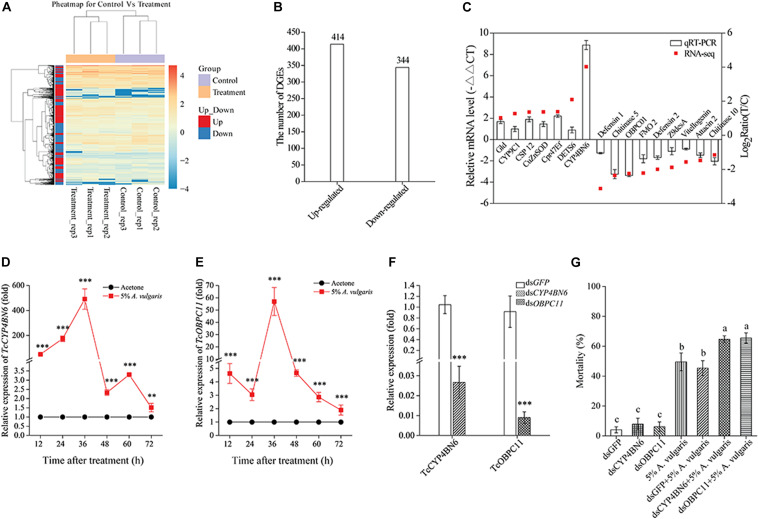
**(A)** Heat map of 758 genes differentially expressed in larvae exposed to 5% *A. vulgaris* essential oil (EO) and control. Rows represent a single gene and columns are comparisons between genes in the exposure treatment and the control. The left-hand column clusters samples (*N* = 6) based on similarity of log10 transformed gene expression. Lighter colors indicate lower levels of differential expression. **(B)** Changes in the gene expression profile between the control and 5% *A. vulgaris* EO exposure groups. **(C)** A comparison of the expression profiles of the selected genes as determined by RNA-sequencing and qRT-PCR. OBP, Odorant binding protein; CYP, Cytochrome P450; Ugt, UDP-glucuronosyltransferase; EST, Esterase; GST, Glutathione *S*-transferase; MRP, Multidrug resistance protein; BG, Beta-glucuronidase; XDH, Xanthine dehydrogenase; FMO, Flavin-containing monooxygenase. Effects of 5% *A. vulgaris* EO exposure on the accumulation of *TcCYP4BN6* **(D)** and *TcOBPC11* **(E)** in 20-day-old larvae of *T. castaneum*. Acetone treatment represents the negative control group, which has been ascribed an arbitrary value of 1. The 12, 24, 36, 48, 60, and 72 h are six different test time points following EO treatment. **(F)** RNAi-mediated gene silencing. Twenty-day-old larvae were used for dsRNA injection. RNA was extracted and quantified by qRT-PCR at fifth day. Control beetles were injected with the same amount of green fluorescent protein (GFP) dsRNA. **(G)** Effect of *TcCYP4BN6*/*TcOBPC11* silencing by injection of ds-*TcCYP4BN6*/ds-*TcOBPC11* on toxicity of EO to 20-day-old larvae. Larvae injected with ds-*TcCYP4BN6*/ds-*TcOBPC11*/ds-*GFP* only served as untreated controls. Following injection with ds-*TcCYP4BN6*/ds-*TcOBPC11*/ds-*GFP*, larvae were treated with EO for 72 h. Data shown are mean ± SE (*n* = 3). An asterisk above bars indicated significant differences in the mRNA expression among the control and treatments (**P < 0.01, ***P < 0.001; Student’s *t*-test). Different letters above bars indicate significant differences (*P* < 0.05) according to LSD multiple comparison tests.

To confirm the significant differences in gene expression between the EO treatment and control groups, a likelihood ratio test was conducted based on the FPKM-derived read count. A reliable statistical analysis was applied to genes with an FPKM value ≥ 2 in at least one of the two biological replicates to minimize the identification of false positives and negatives. It is worth noting that these statistical significance tests were based on expected sampling distributions. To identify unigenes potentially involved in the response of *T. castaneum* larvae to *A. vulgaris* EO, the FPKM values of unigenes were compared between libraries. All three biological replicates of both control and EO treatment groups showed a high correlation, based on cluster analysis (Figure 3A), and *A. vulgaris* EO treated samples formed a distinct cluster separate from the control samples (Supplementary Figure S1C). Furthermore, to confirm which of the genes were differentially expressed to a significant degree between the treatment and control groups, thresholds of FC ≥ 2 and FDR ≤ 10^–3^ were used. Based on these criteria, 758 DEGs were identified. Of these, 344 DEGs were up-regulated and 414 DEGs were down-regulated in EO treated samples compared with control samples (Figure 3B and Supplementary Table S3). To validate the quality of RNA-Seq results, we verified the expression of 16 randomly selected DEGs by qRT-PCR. The expression profiles of these DEGs were similar between qRT-PCR and BGISEQ data, thus confirming the validity of RNA-Seq data (Figure 3C).

### Validation of RNA-Seq Data by qRT- PCR

To confirm the quality of the transcriptome data and DEG results, we selected and evaluated seven up-regulated and nine down-regulated genes with qRT-PCR. The results of qRT-PCR were consistent with RNA-Seq data but with some quantitative variation in the regulation range. In short, qRT-PCR analysis confirmed the directional change in gene expression detected in DEG analysis (Figure 3C).

### Induction of *TcCYP4BN6* and *TcOBPC11* Expression by *A. vulgaris* EO

To determine whether the expression of *TcCYP4BN6* and *TcOBPC11* could be induced by 5% *A. vulgaris* EO, the transcript levels of these two genes were detected by using qRT-PCR after 20-day-old larvae treated with EO at different time points. Interestingly, the transcript levels of *TcCYP4BN6* and *TcOBPC11* were both upregulated in response to 5% *A. vulgaris* EO exposure from 12 to 72 h, and they displayed similarly upregulation patterns (Figures 3D,E). Compared with the acetone controls, *TcCYP4BN6* and *TcOBPC11* were significantly induced by EO, both reaching maximum expression at 36 h with transcript levels of 491.49 ± 81.43- and 57.00 ± 11.38-fold, respectively, their expression then gradually returned to normal levels at 72 h (Figures 3D,E).

### Effect of *TcCYP4BN6* and *TcOBPC11* Silencing on *T. castaneum* Response to *A. vulgaris* EO

To further determine the effects of *TcCYP4BN6* and *TcOBPC11* on insect response to 5% *A. vulgaris* EO, RNAi was used to knock down these two genes. On the fifth day after injection of dsRNAs, qRT-PCR was performed to investigate the gene expression of *TcCYP4BN6* and *TcOBPC11* in *T. castaneum* (Figure 3F). Compared to the control, transcript levels of *TcCYP4BN6* and *TcOBPC11* were significantly decreased by 97.3 and 99.1% in *T. castaneum*, respectively, whereas injection of ds-*GFP* had no significant effect. Hence, RNAi effectively suppressed the expression of *TcCYP4BN6* and *TcOBPC11* in *T. castaneum*. Mortality of the 20-day-old larvae injected with ds-*GFP*, ds-*TcCYP4BN6*, or ds-*TcOBPC11* and exposed to the EO was shown in Figure 3G. Obviously, RNAi against *TcCYP4BN6* or *TcOBPC11* both significantly enhanced insecticidal activity of EO, whereas injection of ds-*GFP* did not (Figure 3G). After exposure to 5% *A. vulgaris* EO, mortality of *T. castaneum* was 45.33% ± 4.98%, 64.67% ± 2.33%, and 65.50% ± 3.50% when injected with ds-*GFP*, ds-*TcCYP4BN6*, or ds-*TcOBPC11*, respectively. Compared to the treatment with 5% *A. vulgaris* EO alone, mortality of *T. castaneum* was significantly increased by 16% or 17% after injection with ds-*TcCYP4BN6* or ds-*TcOBPC11* and treated with EO (Figure 3G). Our results clearly indicated that injection with ds-*TcCYP4BN6* or ds-*TcOBPC11* and exposure to the 5% *A. vulgaris* EO resulted in higher mortality than treatment with the EO alone. These results suggest that *TcCYP4BN6* or *TcOBPC11* may play a vital role in response to *A. vulgaris* EO in *T. castaneum*.

## Discussion

In this paper, the contact activity and insecticidal effect of *A. vulgaris* EO on *T. castaneum* larvae, as assessed following biochemical and comparative RNA-Seq analyses, were characterized and investigated. A pattern of distinct dose-dependence contact activity of *A. vulgaris* EO were detected against *T. castaneum* larvae, consistent with previous studies (Wang et al., 2006). However, we found that the LC50 values were 6.14, 5.11, and 4.77% *A. vulgaris* EO at 24, 48, and 72 h, respectively, revealing there wasn’t apparent time-dependent effect. Actually, as shown in Figures 1C,D, the enzyme activities of CYP and CarE in the larva showed a corresponding pattern of increase at 36 h, but the mortality did not appear time-dependent during this period even at a later time, which may suggest that this degree of induced enzyme activity was not sufficient to cause the change in mortality. This similar phenomenon was also reported in *Apis mellifera* and *Myzus persicae* (Carvalho et al., 2013; Czerniewicz et al., 2018). Meanwhile, to prevent oxidative damage, insects employ detoxifying enzymes, such as CYPs and CarEs, for the metabolism of plant secondary metabolites (Pan et al., 2016). This suggests that the CYPs and CarEs participate in the response of *T. castaneum* larvae to *A. vulgaris* EO. It is possible that the death of *T. castaneum* larvae after *A. vulgaris* EO treatment is caused by the disruption of CYP and CarE homeostasis in insects.

RNA-Seq results showed significant up-regulation of 414 DEGs and significant down-regulation of 344 DEGs in *A. vulgaris* EO treated larvae (Supplementary Table S3). GO enrichment analysis revealed that 142, 99, and 198 DEGs were classified into 37 functional sub-categories in the cellular component, molecular function, and biological process categories, respectively (Figure 2A). KEGG analysis revealed that 511 DEGs were enriched in 262 biological pathways (Supplementary Table S2). Among these groups, we discussed the regulation of biological processes, response to stimuli, antigen processing and presentation, and drug metabolism in further detail, as these are most likely associated with the influence of *A. vulgaris* EO on *T. castaneum* larvae. Exposure to *A. vulgaris* EO promoted the expression of *copper-and zinc-containing superoxide dismutase* (*CuZnSOD*) and *heme peroxidase* (*HPX*); both these genes were simultaneously assigned to the response to stimulus, regulation of biological processes, antioxidant activity, developmental processes, and reproduction GO terms (Table 2 and Supplementary Tables S3, S4). Typically, reactive oxygen species (ROS), including superoxide anion and hydrogen peroxide (H_2_O_2_), produced in cells during metabolism, play pivotal roles in the innate immunity of insects as potent pathogen-killing agents (Bahia et al., 2013; Deng and Zhao, 2014). In addition, the expression of *CuZnSOD* is up-regulated immediately after the exposure of *Brachionus calyciflorus* to H_2_O_2_ treatment, and this increased expression promotes resistance to oxidative stress (Van Raamsdonk et al., 2009; Zhang et al., 2013). Under normal growth conditions, the *dual oxidase* (*Duox*) gene is responsible for ROS generation, which maintains microorganism homeostasis in the fruit fly gut (Kim and Lee, 2014). Nevertheless, higher ROS levels caused by overexpression of *Duox* disrupt cellular function and structure, leading to severe oxidative damage to DNA, RNA, proteins, and lipids (Zhu et al., 2016). These data indicate that the up-regulation of the *CuZnSOD* in EO treated larvae was accompanied by a response to excessive ROS disturbance, further suggesting that *A. vulgaris* EO affects antioxidative stress responses through crosstalk with antioxidant activities of *T. castaneum* larvae. Interestingly, antioxidant activities are also referred to development and reproduction (Taracena et al., 2015; Deng et al., 2016). In *Anopheles gambiae*, the expression of *HPX15*, which encodes as an active peroxidase that functions in reproductive organs to limit oxidative stress, was highly up-regulated. Peroxidases prevent cellular damage caused by free hydroxyl radicals through catalyzing the reduction of H_2_O_2_ to water, implying that *HPX15* is required to maintain fertility (Shaw et al., 2014). These results indicate that the up-regulation of *HPX in T. castaneum* larvae after *A. vulgaris* EO treatment is a protective mechanism that prevents damage to reproductive organs or cells involved in fertility. Furthermore, in the transcriptome data, we found 28 DEGs associated with the lysosome pathway, which is correlated with the likely mode of action of *A. vulgaris* EO in insects. Among these 28 DEGs, 12 were involved in development and reproduction in *T. castaneum* (Table 3). The Cat and *Lipase* genes, which play a significant role in development and reproduction, were significantly down-regulated. For example, *CatB/CatB*-like and *CatL/CatL* precursor participate in metamorphosis by controlling tissue remolding and larval fat body decomposition in *Delia radicum* and *Sarcophaga peregrina*, respectively (Takahashi et al., 1993; Hegedus et al., 2002). Additionally, both *CatB/CatB-*like and *CatL/CatL* precursors are thought to be vital for embryogenesis in mosquito via the degradation of yolk protein (Cho et al., 1999; Uchida et al., 2001). Furthermore, tissue immunohistochemistry indicates that *CatB* participates in the embryonic degradation of yolk protein and fat body histolysis in *Helicoverpa armigera* (Zhao et al., 2005; Yang et al., 2006). In this study, the *CatL* precursor gene was significantly down-regulated after *A. vulgaris* EO treatment. Silencing of *CatL* precursor by RNAi leads to 100% immortality in insects and the absence of vitellogenic egg chambers in 40% of *T. castaneum* eggs (Dönitz et al., 2014). It is possible that EO treatment decreases the activity of CAT enzymes, along with low spawning and insect death. Furthermore, in this paper, the expression level of *Lipase-1 like* was dramatically down-regulated after *A. vulgaris* EO treatment. Previously, knockdown of *Lipase-1 like* resulted in 100% previtellogenic egg chamber orientation in red flour beetle (Dönitz et al., 2014). In addition, *Lipase-3* and *Lipase-3 like* were dramatically down-regulated in the EO treatment group. Furthermore, knockdown of *Lipase-3* reduced the hemolymph lipid concentration, which most likely disturbed lipolysis, leading to eclosion defects in beetles (data not shown). These consequences suggest that *A. vulgaris* EO directly affected lipase biosynthesis in *T. castaneum*. In addition to *Spp38*, two members of the *serine protease* (*Sp*) gene family were notably down-regulated, and 10 members were observably up-regulated in response to *A. vulgaris* EO treatment (*Q*-value ≤ 0.05; Table 3 and Supplementary Table S3). The *SP* genes play a significant role in multiple physiological processes including innate immunity, stress responses, reproduction, and development (Gorman et al., 2000; Zou et al., 2006; Wang et al., 2016). In a previous study, transcript levels of *Sp3*, *Sph42*, and *Sp49* expression levels were elevated after saline or *Escherichia coli* injection, and pathogen challenge further enhanced the expression of *Sp1*, *Sp2*, *Sp6*, and *Sp41* genes in *Apis mellifera* (Zou et al., 2006). Ulteriorly, RNAi against larvae *Sph115* causes developmental arrest with 60% lethality in *T. castaneum*, and knockdown of *Spp2c* in silkworm embryos dramatically reduces the degradation rate of residual yolk proteins on embryonic day 10, further resulting in the suspension of embryogenesis (Dönitz et al., 2014). Moreover, knockdown of *Spp163* or *Spp123* in *T. castaneum* pupae leads to the formation of embryonic tissues without a cuticle, whereas RNAi of *Sph111* causes musculature defects in 30% of larvae (Dönitz et al., 2014). These outcomes indicate that the SP signaling pathway is activated and amplified by *A. vulgaris* EO, which affects stress and immune responses, development, and reproduction in *T. castaneum*. Notably, the loss of activity of these SP proteins in *T. castaneum* larvae was at least partially compensated by up-regulation of the expression of the other primary *Sp* genes (Table 3 and Supplementary Table S4). This result was similar to the SP inhibitor feeding experiment, in which the expression of some *Sp* genes was significantly down-regulated, whereas that of other *Sp* genes was up-regulated, as part of an integrated compensation response in *T. castaneum* (Oppert et al., 2010; Perkin et al., 2017). In total, these data imply that large-scale *Sp* gene expression patterns were dramatically increased when the red flour beetle larvae were exposed to *A. vulgaris* EO, which amplified the SP signaling pathways, ultimately regulating multiple physiological functions in *T. castaneum.* Furthermore, 14 of the DEGs involved were identified in the antigen processing and presentation pathway (Supplementary Table S5). In addition to the seven members of *Lipases* discussed above, these DEGs included four *HSPs* (*HSP68a*, *HSP68b*, *HSP70a*, and *HSP70b*), which were up-regulated after *A. vulgaris* treatment. HSPs are rapidly synthesized in response to various environmental stressors, including heat shock, cold shock, pesticide application, and heavy metal exposure (Huang and Kang, 2007; Rinehart et al., 2007; Shu et al., 2011; Sun et al., 2014; Yang et al., 2016). The expression of *HSP70* is significantly induced in *Drosophila melanogaster*, *Nilaparvata lugens*, *Lymantria dispar*, and *Apolygus lucorum* to increase resistance against applied pesticides (Mrdaković et al., 2016; Sun et al., 2016; Lu et al., 2017). Thus, we speculate that DEGs encoding HSPs participate in the response of *T. castaneum* to *A. vulgaris* EO.

When comparing the control and EO treatment group, RNA-Seq data revealed a large number of multiple metabolic detoxification enzymes in *T. castaneum* larvae (Figure 2B and Table 4). Cell metabolism detoxification processes are divided into four phases (phases 0–III). In phase 0, the uptake of xenobiotics is facilitated by membrane transport proteins. In phase I, CYP enzymes perform oxidation-reduction reactions, and CarE/EST enzymes are involved in the hydrolysis of ester bonds, leading to the introduction of a polar group in the toxic molecules (Montella et al., 2012; Schama et al., 2016). Meanwhile, phase II involves many cellular defense enzymes such as GSTs and Ugts mediated conjugation reactions of phase I metabolites with one of several endogenous molecules to form water-soluble products (Ando et al., 2000; Glisic et al., 2015). Finally, in phase III, reaction products of the previous step are transferred out of the cell by transport proteins (Vieira-Brock et al., 2013). In this article, genes encoding four CSP proteins (CSP11, 12, 17, and 20) and five OBPs (OBP10, 11, 12, 17, and C02) were up-regulated in EO treated larvae. The high expression of *CSPs* and *OBPs* was referred to drug metabolism because of their ability to bind lipophilic compounds, indicating that CSPs and OBPs bind to hydrophobic xenobiotics in phase 0 of the cellular detoxification process in insects (Liu et al., 2010, 2014; Bautista et al., 2015). We speculate that *CSPs* and *OBPs* are up-regulated in beetles to create resistance to *A. vulgaris*. In response to *A. vulgaris* EO treatment, *CSP8* and *OBPC01* were down-regulated, further disrupting the homeostasis of xenobiotics *in vivo*. Moreover, the expression of *FMOs*, *ESTs*, and *XDHs* as well as that of *CYPs* was also dramatically affected in EO treated larvae (Table 4). Furthermore, genes encoding CYP4BN1, CYP6BQ7, CYP6A2, CYP351A2, CYP9Z2, CYP9AC1, CYP6BK11, and CYP6A14 were up-regulated in the EO treatment group. The CYP unigenes were significantly up-regulated in the EO treated larvae, indicating that these genes are involved in *A. vulgaris* EO activation and detoxification pathways, which catalyze intracellular redox reactions (Janmohamed et al., 2001). However, six *CYP* genes (*CYP4BN5 CYP4Q1*, *CYP351A3*, *CYP4C1*, *CYP6A20*, and *CYP349A1*) were down-regulated, which reduced the cellular metabolic activity, thus causing insect death (Dönitz et al., 2014). Furthermore, like *CYPs*, the expression of *FMOs*, which are related to NADPH oxidase activity (N-oxidation), decreased after treatment with *A. vulgaris* EO. FMOs can catalyze the conversion of toxic pyrrolizidine alkaloids (PAs) into the non-toxic N-oxide form, and can also regulate the detoxification of nucleophilic nitrogen- and sulfur-containing xenobiotics in vertebrates (Hartmann, 2009). The *CYP* and *FMO* family genes, which were expressed to low levels, were involved in drug metabolism, indicating a correlation between these genes in the detoxification process (Chung et al., 2000; Zhu et al., 2015). Additionally, genes encoding CarEs (*α-EST5* and *α-EST2*) were also up-regulated by EO treatment, suggesting that these genes could participate in catalyzing the hydrolysis of various xenobiotics in *A. vulgaris* EO (Table 4). In fact, our biochemical analysis showed that *A. vulgaris* EO caused a pronounced increase in CarE activity.

**FIGURE 4:**
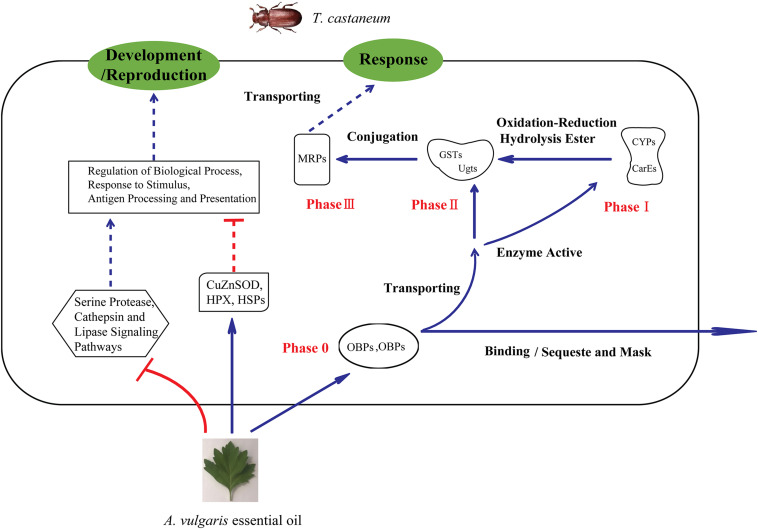
Schematic of the mechanism of insect response to stimulation by *A. vulgaris* essential oil. OBPs, Odorant-binding proteins; CSPs, chemosensory proteins; CYPs, cytochrome P450 monooxygenases; CarE, carboxylesterase; Ugt, UDP-glucuronosyltransferase; GST, Glutathione *S*-transferase; MRP, multidrug resistance protein; (HPX), heme peroxidase; HSP, heat shock protein; CuZnSOD, copper-and zinc-containing superoxide dismutase.

Genes encoding enzymes belonging to phase II, including BGs, GSTs, and Ugts, were also up-regulated after EO treatment. In a previous study, the activity of BGs was increased upon low-level exposure to an organophosphorus insecticide (Ueyama et al., 2010). This indicates that exposure to *A. vulgaris* EO caused the down-regulation of *BG* expression in *T. castaneum* larvae, suggesting that *A. vulgaris* EO represses phase II genes by regulating *BG* expression. Generally, GSTs directly detoxify organophosphates and organochlorine through the conjugation of these electrophilic compounds with the thiol group of reduced GSH, thus rendering the products more water-soluble and excretable than the non-GSH conjugated substrates (Aravindan et al., 2014). Additionally, GSTs possibly have no direct role in the metabolism of pyrethroids, but they could detoxify lipid peroxidation products induced by insecticides, which also demonstrated in coffering resistance (Vontas et al., 2001). In the current study, three *GST* genes (*GSTs6*, *GSTs7*, and *GSTe4-like*) were up-regulated in EO samples (Table 4), indicating that a growing number of toxic intermediates were transformed into innocuous substances by conjugation with GSH which also caused various endogenous molecules like sugars and glutathione pathway were expressed to conjugate xenobiotics. Moreover, two *GST* genes (*GSTe7* and *GSTe8*) were down-regulated in EO samples. It is possible that redundant components could bind to the enzyme site, thus disturbing enzyme activity. The conversion of conjugated xenobiotics to innocuous substances possibly damaged the enzymes to the point of no recovery. Both induction and inhibition of *GST* expression in response to certain plant secondary metabolites has been previously reported (Mostofa et al., 2014; Balyan et al., 2015). Notably, Ugts participate in phase II reactions, similar to GSTs, and catalyze the transfer of glucuronic acid to lipophilic molecules to further increase the water solubility of the compound for later excretion or sequestration (Okazaki and Katayama, 2003; Crava et al., 2016). Furthermore, Ugts detoxify xenobiotics, including insecticides, and have been linked to insecticide resistance (Lv et al., 2016). In this study, we detected six *Ugts* in EO treated samples, implying that these genes possibly take part in the metabolic activation and detoxification of *A. vulgaris* EO, which accelerates the water solubility of compounds. The up-regulation of *multidrug resistance proteins* (*MRPs*) was also detected in EO treated larvae; MRPs are phase III reactions and utilize the energy derived from ATP hydrolysis to translocate a variety of physiological metabolites and xenobiotics (Table 4) (Vieira-Brock et al., 2013). It is likely that insects increase the expression of *MRP* genes to enhance the efficiency of xenobiotic compound excretion or degradation. This is the first report of evidence demonstrating that cellular detoxification genes of phases 0–III, including *OBPs*, *CSPs*, *CYPs*, *ESTs*, *FMOs*, *GSTs*, *Ugts*, and *BG*, are involved in the response of *T. castaneum* to *A. vulgaris* EO (Figure 4).

Further, two DEGs (*TcOBPC11* and *TcCYP4BN6*) which were likely involved in the response of *T. castaneum* to *A. vulgaris* EO were performed for EO stimulation and induction studies. Interestingly, in our study, after *A. vulgaris* EO treatment, the transcripts of *TcOBPC11* and *TcCYP4BN6* were significantly induced. Knockdown *TcOBPC11* or *TcCYP4BN6*, the mortality of *T. castaneum* beetles was significantly increased compared with the controls (Figures 3D,E), indicating that *TcOBPC11* or *TcCYP4BN6* were involved in response to *A. vulgaris* EO in *T. castaneum*. Currently, studies examining the canonical biological functions of OBPs have focused on their activity within insect chemosensory systems, trying to understand their roles in detecting and recognizing environmental chemical stimuli (Sánchez-Gracia et al., 2009). Generally, the expression of insect *OBPs* associated with xenobiotic resistance is often inducible by non-toxic compounds, host allelochemicals and synthetic insecticides (Bautista et al., 2015; Xuan et al., 2015). Interestingly, such induction pattern of *TcOBPC11* was reminiscent of *SlituOBP9*, the expression level of which was obviously promoted by chlorpyrifos in *Spodoptera litura* (Lin et al., 2018). Similar results have also been reported for *P. xylostella PxylOBP13*, *T. castaneum TcOBPC01*, which the transcript levels of these genes were upregulated following permethrin, carbofuran/dichlorvos treatment, respectively (Bautista et al., 2015; Xiong et al., 2019a). Thus we speculate that the activity structure of some compositions in *A. vulgaris* EO is similar to these insecticides. Simultaneously, in insects, *CYP* genes play an essential role in detoxifying exogenous compounds, including plant toxins and insecticides, and their upregulation can result in increased levels of CYP proteins and CYP enzyme activities (Zhu et al., 2010). The increased levels of *TcCYP4BN6* expression observed during the response to *A. vulgaris* EO are ostensibly consistent with a role for *TcCYP4BN6* in EO metabolism in *T. castaneum* (Figure 3D). The observed induction pattern of *TcCYP4BN6* is reminiscent of those of *SinvCYP4AB2* and *SinvCYP4G15* and those of *SinvCYP6A1* and *SinvCYP6B1* in *Solenopsis invicta*, and expression of these genes has been shown to be promoted by fipronil (Zhang et al., 2016). Similar results have also been reported for *M. domestica*, in which the transcript levels of *MuscaCYP4G2* and *MuscaCYP6A38* were upregulated following permethrin treatment (Zhu et al., 2008). Furthermore, 22 CYPs unigenes in *Melaleuca alternifolia* EO-fumigated *Sitophilus zeamais* were significantly up-regulated (Liao et al., 2016). These results indicate that different types of insecticides, EOs exert some common effects on the upregulation of the expression of insecticide metabolism-related *CYP* genes. Notably, *TcOBPC11* and *TcCYP4BN6* were both significantly increased with exposure to 5% *A. vulgaris* EO at 12–72 h, both reaching maximum expression at 36 h, then gradually returned to normal levels at 72 h (Figures 3D,E). Perhaps it takes 72 h after exposure to EO to recruit multiple susceptibility genes in a variety of defense mechanisms (Liao et al., 2016). Moreover, a time-dependent induction of susceptibility genes is typically observed after 12 h (Zhu et al., 2008). In particular, hundreds of genes are differentially expressed during the 48–60 h period following *Bemisia tabaci* fungal infection (Xia et al., 2013). These studies combined with our present results (Figures 3D,E) strongly imply that this delay in response could be due to the time needed for the exogenous toxic molecules to penetrate through the cuticle of insects (Liu et al., 2014). Therefore, different *CYP* and *OBP* genes could be involved in different exposure times to protect various tissue specific physiological pathways.

RNAi has emerged as a powerful tool for researching gene functions within a wide range of both multicellular organisms and unicellular (Meister and Tuschl, 2004; Kim et al., 2015). Using dsRNA injection, we found that RNAi against *TcOBPC11* or *TcCYP4BN6* significantly increased mortality of *T. castaneum* when exposed to 5% *A. vulgaris* EO, indicating that *TcOBPC11* or *TcCYP4BN6* might be play a vital role during the response to *A. vulgaris* EO treatments (Figure 3G). Exposure to *A. vulgaris* EO increased the expression levels of *TcOBPC11*, but knocking this gene down prior to EO exposure led to even higher mortalities, which implied that the roles of this genes might be protective because *TcOBPC11* is acted as buffers that have a high binding affinity with *A. vulgaris* EO. Increasing the expression of *TcOBPC11* would decrease the toxicity of EO through high binding affinity, consequently sequestering and masking toxic molecules (Pelosi et al., 2017; Lin et al., 2018). Simultaneously, our result showed similar expression pattern and biological assay have been found in *TcCYP4BN6* after *A. vulgaris* EO treatment, which implying that the roles of this CYP protein in *T. castaneum* are likely be protective. *TcCYP4BN6* is known as members of the CYP family that are associated with insecticide metabolic detoxification (Schama et al., 2016), and increasing the expression of these *CYP* genes would be expected to decrease the toxicity of *A. vulgaris* EO through oxidation–reduction reactions that occur during the metabolism of toxic molecules (Wang R. L. et al., 2015). In our current study, *A. vulgaris* EO induced overexpression of *TcOBPC11* or *TcCYP4BN6* in *T. castaneum*. Additionally, RNAi mediated silencing of *TcOBPC11* or *TcCYP4BN6* significantly increased mortality of *T. castaneum* larvae exposed to EO. These results strongly reveal that *TcOBPC11* or *TcCYP4BN6* might play an important role in *A. vulgaris* EO metabolic detoxification in *T. castaneum*, which further confirms our RNA-seq result.

## Conclusion

In this paper, we examined the contact toxicity of *A. vulgaris* EO on *T. castaneum;* its effects on the activities of two types of insect detoxification enzymes were also evaluated. The results suggested that *A. vulgaris* EO has the potential to be used as a natural insecticide. More importantly, this is the first report of a comprehensive transcriptome analysis of *T. castaneum* to: (1) identify genes and pathways involved in the response to *A. vulgaris* EO exposure, and (2) investigate the underlying molecular mechanism of insecticidal activity against *T. castaneum* larvae. Enzyme activities of CarEs and CYPs were dramatically increased in EO treated larvae. RNA-seq results confirmed that *OBPs*, *CSPs*, *P450s*, *GSTs*, *UGTs*, and *MRPs* participate in the metabolism of *A. vulgaris* EO. (3) In addition, our bioassay results also supported that *OBPs* and *P450s* might play an important role in *A. vulgaris* EO metabolic detoxification in *T. castaneum*. Thus, our results will not only accelerate studies investigating the mechanisms underlying the insecticidal effect of plant EO on insect pests but also facilitate the development of natural novel insecticides.

## Data Availability Statement

Raw sequence reads were saved as FASTQ files and deposited in the NCBI Sequence Read Archive (SRA) database under the following accession numbers: control: SRR7646221, SRR7646223, and SRR7646224; EO treatment: SRR7646222, SRR7646225, and SRR7646226.

## Author Contributions

SG and KZ designed the experiments. LW, GW, and WX carried out the experiments. YL, YZ, and AG analyzed the experimental results. BL wrote the manuscript. All authors contributed to the article and approved the submitted version.

## Conflict of Interest

The authors declare that the research was conducted in the absence of any commercial or financial relationships that could be construed as a potential conflict of interest.

## Footnotes

1 http://www.seq50.com/en/

2 http://hannonlab.cshl.edu/fastx_toolkit/

3 http://www.beetlebase.org/

4 http://www.bioconductor.org/packages/release/bioc/html/NOISeq.html

5 http://www.geneontology.org/

6 http://www.genome.jp/keg

7 http://kobas.cbi.pku.edu.cn

## Supplementary Material

FIGURE S1 **(A)** Length distribution of the gene detected by RNA-sequencing. The *x*-axis and *y*-axis indicate the length of the genes and the number of genes, respectively. **(B)** FPKM statistic of the control and treatment. The *x*-axis and *y*-axis indicated the FPKM value of the genes and the number of genes, respectively. **(C)** Cluster tree. Distances between the expressed genes were calculated by the Euclidean method. The algorithm of the sum of squares of deviations was used to calculate the distance between samples to construct the cluster tree. *Y*-axis represented the height of the cluster tree. Samples with similar height were clustered together.

FIGURE S2 Percent of coverage representing the percentage of genes that were expressed in each of the two libraries mapped in the red flour beetle (*Tribolium castaneum*) genome. Panels **(A–C)** control groups; **(D,E)**, 5% *Artemisia vulgaris* EO treatment groups. The gene coverage was the percentage of a gene covered by the reads. This value was equal to the ratio of the base number in a gene covered by unique mapping reads to the total base number of that gene. The saturation analyses of all the detected genes. Panels **(G–I)** control groups; **(J–L)** 5% *Artemisia vulgaris* EO treatment groups.

TABLE S1 Primers used for qRT-PCR analysis and dsRNA synthesis in this study.

TABLE S2 Pathway enrichment analysis of differentially expressed genes (DEGs) and all the detected genes.

TABLE S3 A set of 758 differentially expressed genes (DEGs) between the 5% *Artemisia vulgaris* treatment and control groups.

TABLE S4 Differentially expressed genes (DEGs) involved in response to stimuli.

TABLE S5 Differentially expressed genes (DEGs) related to antigen processing and presentation.
