# Supplementary figures and images for: Insecticidal Activity of Artemisia vulgaris Essential Oil and Transcriptome Analysis of Tribolium castaneum in Response to Oil Exposure
Source: Front Genet. 2020 Jun 25;11:589. doi: 10.3389/fgene.2020.00589 (PMC7330086; doi:10.3389/fgene.2020.00589)

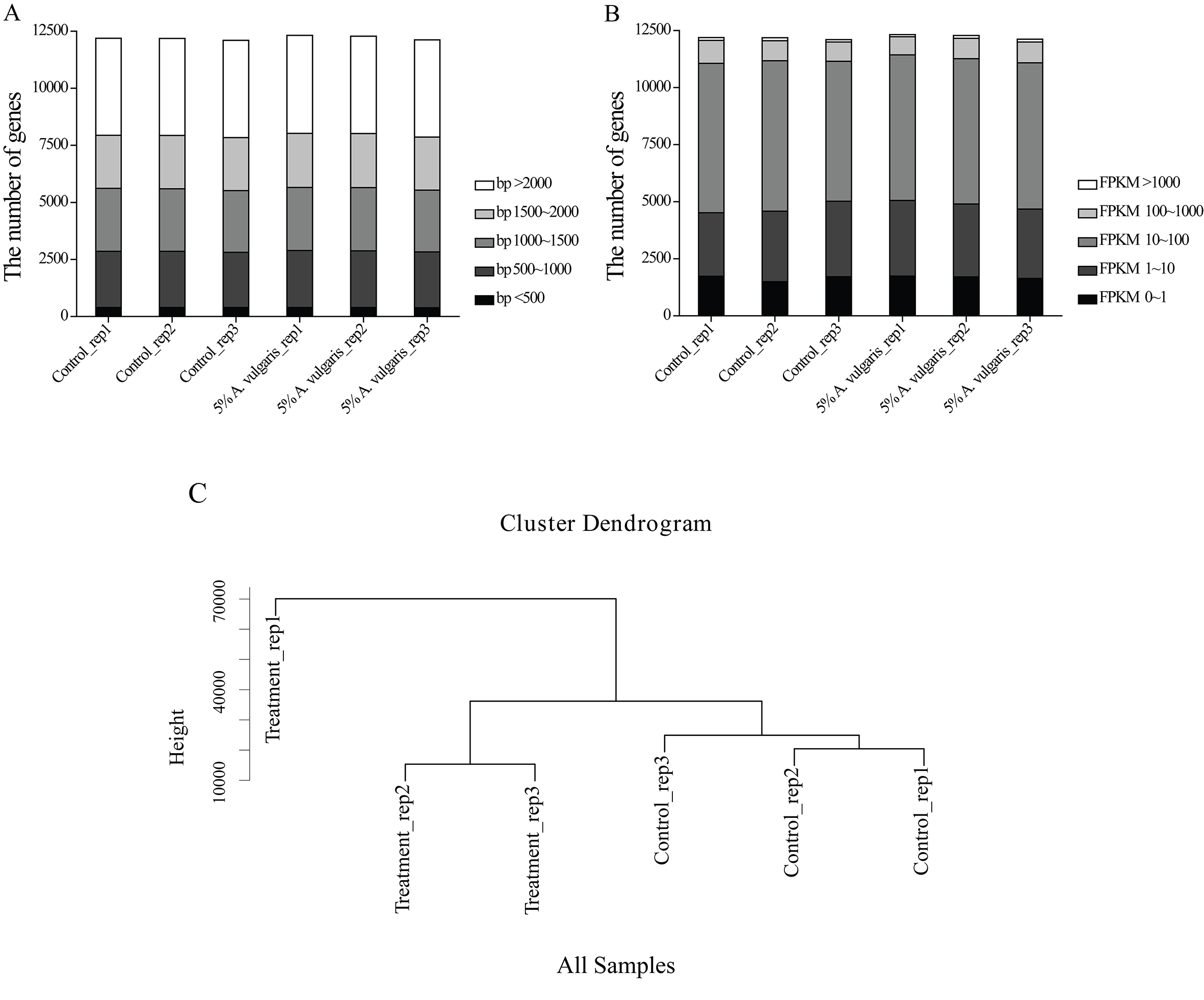

Supplement: FIGURE S1 — (A) Length distribution of the gene detected by RNA-sequencing. The x-axis and y-axis indicate the length of the genes and the number of genes, respectively. (B) FPKM statistic of the control and treatment. The x-axis and y-axis indicated the FPKM value of the genes and the number of genes, respectively. (C) Cluster tree. Distances between the expressed genes were calculated by the Euclidean method. The algorithm of the sum of squares of deviations was used to calculate the distance between samples to construct the cluster tree. Y-axis represented the height of the cluster tree. Samples with similar height were clustered together. [file Image_1.TIF]

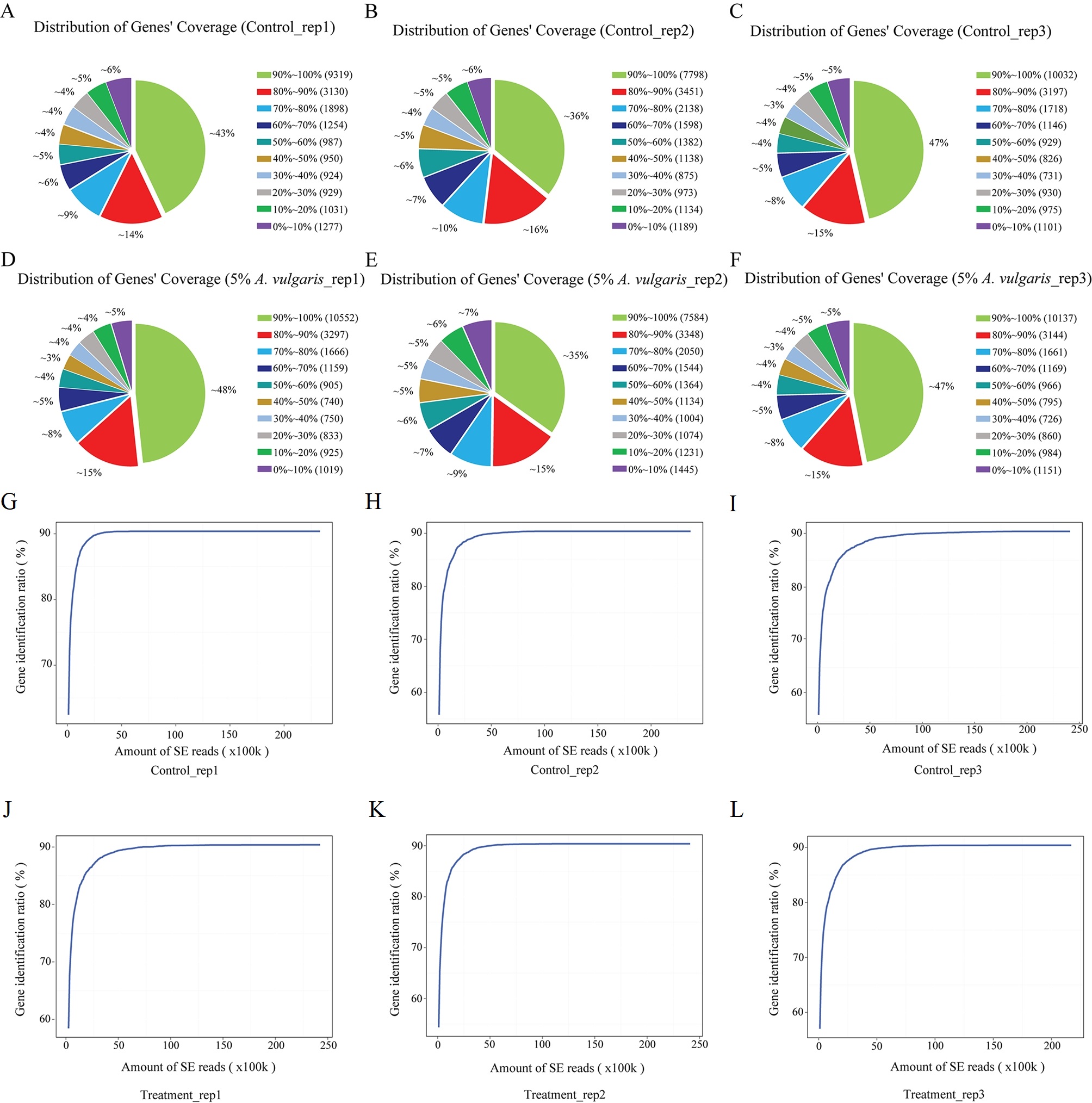

Supplement: FIGURE S2 — Percent of coverage representing the percentage of genes that were expressed in each of the two libraries mapped in the red flour beetle (Tribolium castaneum) genome. Panels (A–C) control groups; (D,E), 5% Artemisia vulgaris EO treatment groups. The gene coverage was the percentage of a gene covered by the reads. This value was equal to the ratio of the base number in a gene covered by unique mapping reads to the total base number of that gene. The saturation analyses of all the detected genes. Panels (G–I) control groups; (J–L) 5% Artemisia vulgaris EO treatment groups. [file Image_2.JPEG]
