# Supplementary material for: Insecticidal Activity of Artemisia vulgaris Essential Oil and Transcriptome Analysis of Tribolium castaneum in Response to Oil Exposure
Source: Front Genet. 2020 Jun 25;11:589. doi: 10.3389/fgene.2020.00589 (PMC7330086; doi:10.3389/fgene.2020.00589)
Supplement: TABLE S1 — Primers used for qRT-PCR analysis and dsRNA synthesis in this study. [file Table_1.docx]

**Supplementary Table S1** Primers used for qRT-PCR analysis and dsRNA synthesis in this study

| Gene symbol | Sense primer | Anti-sence primer | Protein | Remarks |
| --- | --- | --- | --- | --- |
| LOC656559 | GCAAATTGGTACTCTTGTCGATG | TATTAGAATTTTCGTCGCGGTG | Gld | qRT-PCR |
| LOC657454 | AGAACTCTACAATGCCTTCCCTG | AATCTTCGGTGGTTGACAAAATG | CYP9AC1 | qRT-PCR |
| LOC661270 | TAGTCCGGACGGACAAGAAT | CCAACTCGTTCCACCAATCA | CSP12 | qRT-PCR |
| LOC660957 | CTACCAAAGCGACCTCTACGA | CTTCCTCCACTTCTATGTTCCCC | CuZnSOD | qRT-PCR |
| LOC658557 | TGTATTTGCCCCCTGTTAGC | GCCGATTTCCTGTTGACTGA | Cpr47Ef | qRT-PCR |
| LOC663364 | TGATTTCGAGACCAAAGCAG | TCCAAGAGAAACTGCCACAG | DETS 6 | qRT-PCR |
| LOC660270 | TTCAAGAATAAAACCAAAGGCG | GTCGTACCCATTGCTGTTTCACA | CYP4BN6 | qRT-PCR |
| LOC662384 | CATCATTCTTCGAAAAACCCAG | ATGATTTAGCTTGATGGAGACGC | Defensin 1 | qRT-PCR |
| LOC641601 | ACTTTGTGGAGGAGCTGAGAC | CGACCCAAAGTTGCAAACCA | Chitinase 5 | qRT-PCR |
| LOC664598 | AACCCAAACTGAAAAACCACG | TGACCTTCCTCAAGCAAACGA | OBPC01 | qRT-PCR |
| LOC103313826 | GTGGTACTTGGGTTTACACCGATA | ACAACTTCCTGTGACACATAAGATTC | FMO 2 | qRT-PCR |
| LOC656629 | GTTGCCCTTTTCTGCATCTTC | GGAATGGTTTAGCTTGACGGAG | Defensin 2 | qRT-PCR |
| LOC658103 | CTCAGCCAAGTTAGCAACTTCG | CGGAGAATTTGTGGTGGACG | Z9desA | qRT-PCR |
| LOC658755 | TGATGCTTTGCAAAAAATGTG | CGACAAGGCGGTTTCGTAAT | Vitellogenin | qRT-PCR |
| LOC100141947 | CTCCGCCAAACGACCAAA | GGATGCTTCAACGCCGACT | Attacin 2 | qRT-PCR |
| LOC652967 | AGCTACCACTTGTCGTGTGC | TACACCACGTGTGTGCAAAG | Chitinase 10 | qRT-PCR |
| LOC656161 | ATTGTTTTGTGCCTTCTTGCC | GTCCCTTCAGCTTCATTGCTC | OBPC11 | qRT-PCR |
| CB335975 | TCAAATTGATCGGAGGTTTG | GTCCCACGGCAACATAATCT | Rps3 | qRT-PCR |
| LOC660270 | TAATACGACTCACTATAGGG  ACGCTGCTGGCGATGTTAT | TAATACGACTCACTATAGGG  TTACTTTGGACGCTTGGTCTTTT | CYP4BN6 | RNAi |
| LOC656161 | TAATACGACTCACTATAGGG  AGATGATGAACGATGCTGGAGA | TAATACGACTCACTATAGGG  AATTTTTGTTGCGGATGAGACA | OBPC11 | RNAi |
| / | TAATACGACTCACTATAGGG  CGATGCCACCT | TAATACGACTCACTATAGGG  TCGCCCTCG | GFP | RNAi |

Note: Gld, Glucose dehydrogenas; CYP9AC1, Cytochrome P450 9AC1; CSP12, Chemosensory protein 12; CuZnSOD, Copper-and Zinc-containing superoxide dismutase; Cpr47Ef, Cuticular protein 47Ef; DETS 6, DNA-binding protein DETS-6; CYP4BN1, Cytochrome P450 4BN1; OBPC01, Odorant binding protein C01; FMO 2, Flavin-containing monooxygenase 2; Z9desA, Z9 acyl Coenzyme-A desaturase; Rps3, ribosomal protein S3; The letters with a gray undertone are the T7 promoters for dsRNA synthesis.
