# Supplementary material for: Insecticidal Activity of Artemisia vulgaris Essential Oil and Transcriptome Analysis of Tribolium castaneum in Response to Oil Exposure
Source: Front Genet. 2020 Jun 25;11:589. doi: 10.3389/fgene.2020.00589 (PMC7330086; doi:10.3389/fgene.2020.00589)
Supplement: TABLE S2 — Pathway enrichment analysis of differentially expressed genes (DEGs) and all the detected genes. [file Table_2.docx]

**Supplementary Table S2** Pathway enrichment analysis of DEGs and all the detected genes

|  | Pathway | DEGs genes with pathway annotation (511) | All genes with pathway annotation (10027) | *P*-value | *Q*-value | Pathway ID |
| --- | --- | --- | --- | --- | --- | --- |
| 1 | Antigen processing and presentation | 14 (2.74%) | 65 (0.65%) | 3.68E-06 | 0.000964 | ko04612 |
| 2 | Carbohydrate digestion and absorption | 11 (2.15%) | 46 (0.46%) | 1.42E-05 | 0.001478 | ko04973 |
| 3 | Lysosome | 28 (5.48%) | 231 (2.3%) | 1.69E-05 | 0.001478 | ko04142 |
| 4 | Metabolic pathways | 108 (21.14%) | 1518 (15.14%) | 0.000123 | 0.008069 | ko01100 |
| 5 | Amino sugar and nucleotide sugar metabolism | 16 (3.13%) | 121 (1.21%) | 0.000409 | 0.021426 | ko00520 |
| 6 | Metabolism of xenobiotics by cytochrome P450 | 13 (2.54%) | 89 (0.89%) | 0.000537 | 0.023459 | ko00980 |
| 7 | Tyrosine metabolism | 8 (1.57%) | 39 (0.39%) | 0.000653 | 0.024436 | ko00350 |
| 8 | Longevity regulating pathway - multiple species | 11 (2.15%) | 70 (0.7%) | 0.000759 | 0.02487 | ko04213 |
| 9 | Phenylalanine metabolism | 8 (1.57%) | 42 (0.42%) | 0.001095 | 0.030126 | ko00360 |
| 10 | Systemic lupus erythematosus | 7 (1.37%) | 33 (0.33%) | 0.00115 | 0.030126 | ko05322 |
| 11 | Drug metabolism - cytochrome P450 | 12 (2.35%) | 87 (0.87%) | 0.001455 | 0.034656 | ko00982 |
| 12 | Cutin, suberine and wax biosynthesis | 6 (1.17%) | 27 (0.27%) | 0.002012 | 0.038716 | ko00073 |
| 13 | Fatty acid biosynthesis | 5 (0.98%) | 19 (0.19%) | 0.00216 | 0.038716 | ko00061 |
| 14 | Starch and sucrose metabolism | 7 (1.37%) | 37 (0.37%) | 0.002323 | 0.038716 | ko00500 |
| 15 | Glutathione metabolism | 11 (2.15%) | 80 (0.8%) | 0.002325 | 0.038716 | ko00480 |
| 16 | Chemical carcinogenesis | 12 (2.35%) | 92 (0.92%) | 0.002364 | 0.038716 | ko05204 |
| 17 | Steroid hormone biosynthesis | 9 (1.76%) | 59 (0.59%) | 0.002783 | 0.042884 | ko00140 |
| 18 | ABC transporters | 11 (2.15%) | 83 (0.83%) | 0.003123 | 0.045452 | ko02010 |
| 19 | Pentose and glucuronate interconversions | 11 (2.15%) | 84 (0.84%) | 0.003433 | 0.047337 | ko00040 |
| 20 | Arginine and proline metabolism | 8 (1.57%) | 52 (0.52%) | 0.00447 | 0.058554 | ko00330 |
| 21 | Glycosaminoglycan degradation | 6 (1.17%) | 32 (0.32%) | 0.004964 | 0.061936 | ko00531 |
| 22 | Wnt signaling pathway | 15 (2.94%) | 143 (1.43%) | 0.006153 | 0.072397 | ko04310 |
| 23 | Phagosome | 14 (2.74%) | 130 (1.3%) | 0.006355 | 0.072397 | ko04145 |
| 24 | Steroid biosynthesis | 6 (1.17%) | 34 (0.34%) | 0.006761 | 0.073803 | ko00100 |
| 25 | Cholesterol metabolism | 10 (1.96%) | 82 (0.82%) | 0.008537 | 0.088435 | ko04979 |
| 26 | Biotin metabolism | 3 (0.59%) | 9 (0.09%) | 0.008776 | 0.088435 | ko00780 |
| 27 | Aminoacyl-tRNA biosynthesis | 8 (1.57%) | 59 (0.59%) | 0.009659 | 0.093727 | ko00970 |
| 28 | Proteoglycans in cancer | 17 (3.33%) | 180 (1.8%) | 0.010343 | 0.096784 | ko05205 |
| 29 | Rheumatoid arthritis | 7 (1.37%) | 49 (0.49%) | 0.011463 | 0.103563 | ko05323 |
| 30 | Apoptosis | 12 (2.35%) | 113 (1.13%) | 0.01231 | 0.107508 | ko04210 |
| 31 | Peroxisome | 15 (2.94%) | 156 (1.56%) | 0.013276 | 0.1122 | ko04146 |
| 32 | Drug metabolism - other enzymes | 13 (2.54%) | 130 (1.3%) | 0.015085 | 0.11981 | ko00983 |
| 33 | Galactose metabolism | 8 (1.57%) | 64 (0.64%) | 0.015465 | 0.11981 | ko00052 |
| 34 | Pyruvate metabolism | 11 (2.15%) | 103 (1.03%) | 0.015548 | 0.11981 | ko00620 |
| 35 | Glycolysis / Gluconeogenesis | 10 (1.96%) | 92 (0.92%) | 0.018357 | 0.137417 | ko00010 |
| 36 | Complement and coagulation cascades | 5 (0.98%) | 32 (0.32%) | 0.021708 | 0.157988 | ko04610 |
| 37 | Osteoclast differentiation | 7 (1.37%) | 57 (0.57%) | 0.024943 | 0.176624 | ko04380 |
| 38 | Protein digestion and absorption | 12 (2.35%) | 126 (1.26%) | 0.026874 | 0.185291 | ko04974 |
| 39 | Retinol metabolism | 8 (1.57%) | 72 (0.72%) | 0.029449 | 0.197839 | ko00830 |
| 40 | Primary bile acid biosynthesis | 2 (0.39%) | 6 (0.06%) | 0.033912 | 0.216121 | ko00120 |
| 41 | Basal cell carcinoma | 5 (0.98%) | 36 (0.36%) | 0.03445 | 0.216121 | ko05217 |
| 42 | Vitamin digestion and absorption | 7 (1.37%) | 61 (0.61%) | 0.034645 | 0.216121 | ko04977 |
| 43 | Neuroactive ligand-receptor interaction | 14 (2.74%) | 163 (1.63%) | 0.038585 | 0.234762 | ko04080 |
| 44 | Insect hormone biosynthesis | 7 (1.37%) | 63 (0.63%) | 0.040322 | 0.234762 | ko00981 |
| 45 | Porphyrin and chlorophyll metabolism | 7 (1.37%) | 63 (0.63%) | 0.040322 | 0.234762 | ko00860 |
| 46 | Ascorbate and aldarate metabolism | 7 (1.37%) | 65 (0.65%) | 0.046572 | 0.260377 | ko00053 |
| 47 | Autophagy - animal | 12 (2.35%) | 137 (1.37%) | 0.046709 | 0.260377 | ko04140 |
| 48 | Monobactam biosynthesis | 1 (0.2%) | 1 (0.01%) | 0.050962 | 0.27817 | ko00261 |
| 49 | Carbon metabolism | 15 (2.94%) | 187 (1.86%) | 0.054618 | 0.287459 | ko01200 |
| 50 | Citrate cycle (TCA cycle) | 9 (1.76%) | 96 (0.96%) | 0.055475 | 0.287459 | ko00020 |
| 51 | Influenza A | 11 (2.15%) | 126 (1.26%) | 0.056405 | 0.287459 | ko05164 |
| 52 | Toxoplasmosis | 7 (1.37%) | 68 (0.68%) | 0.057053 | 0.287459 | ko05145 |
| 53 | Fluid shear stress and atherosclerosis | 11 (2.15%) | 127 (1.27%) | 0.059064 | 0.289272 | ko05418 |
| 54 | Breast cancer | 8 (1.57%) | 83 (0.83%) | 0.060174 | 0.289272 | ko05224 |
| 55 | Pantothenate and CoA biosynthesis | 3 (0.59%) | 18 (0.18%) | 0.060725 | 0.289272 | ko00770 |
| 56 | Longevity regulating pathway - worm | 9 (1.76%) | 99 (0.99%) | 0.06496 | 0.303584 | ko04212 |
| 57 | Cocaine addiction | 5 (0.98%) | 43 (0.43%) | 0.066047 | 0.303584 | ko05030 |
| 58 | Alcoholism | 9 (1.76%) | 100 (1%) | 0.068336 | 0.30869 | ko05034 |
| 59 | beta-Alanine metabolism | 5 (0.98%) | 44 (0.44%) | 0.071546 | 0.317713 | ko00410 |
| 60 | Ubiquinone and other terpenoid-quinone biosynthesis | 4 (0.78%) | 32 (0.32%) | 0.077669 | 0.339154 | ko00130 |
| 61 | Gastric cancer | 7 (1.37%) | 74 (0.74%) | 0.08208 | 0.352542 | ko05226 |
| 62 | Fatty acid metabolism | 7 (1.37%) | 75 (0.75%) | 0.086781 | 0.366719 | ko01212 |
| 63 | Primary immunodeficiency | 2 (0.39%) | 10 (0.1%) | 0.088946 | 0.369902 | ko05340 |
| 64 | Renin-angiotensin system | 4 (0.78%) | 34 (0.34%) | 0.092647 | 0.379273 | ko04614 |
| 65 | Neomycin, kanamycin and gentamicin biosynthesis | 1 (0.2%) | 2 (0.02%) | 0.099332 | 0.400386 | ko00524 |
| 66 | Purine metabolism | 17 (3.33%) | 242 (2.41%) | 0.111857 | 0.440389 | ko00230 |
| 67 | Hepatocellular carcinoma | 9 (1.76%) | 111 (1.11%) | 0.112619 | 0.440389 | ko05225 |
| 68 | Toll-like receptor signaling pathway | 6 (1.17%) | 66 (0.66%) | 0.11882 | 0.457806 | ko04620 |
| 69 | Platelet activation | 7 (1.37%) | 82 (0.82%) | 0.123815 | 0.466044 | ko04611 |
| 70 | AMPK signaling pathway | 8 (1.57%) | 98 (0.98%) | 0.12621 | 0.466044 | ko04152 |
| 71 | Glycine, serine and threonine metabolism | 4 (0.78%) | 38 (0.38%) | 0.126294 | 0.466044 | ko00260 |
| 72 | Terpenoid backbone biosynthesis | 4 (0.78%) | 40 (0.4%) | 0.144789 | 0.514234 | ko00900 |
| 73 | Other glycan degradation | 4 (0.78%) | 40 (0.4%) | 0.144789 | 0.514234 | ko00511 |
| 74 | Graft-versus-host disease | 1 (0.2%) | 3 (0.03%) | 0.145242 | 0.514234 | ko05332 |
| 75 | Signaling pathways regulating pluripotency of stem cells | 6 (1.17%) | 74 (0.74%) | 0.174721 | 0.607005 | ko04550 |
| 76 | Glycosphingolipid biosynthesis - globo and isoglobo series | 2 (0.39%) | 15 (0.15%) | 0.176078 | 0.607005 | ko00603 |
| 77 | Allograft rejection | 1 (0.2%) | 4 (0.04%) | 0.188815 | 0.637199 | ko05330 |
| 78 | Measles | 5 (0.98%) | 60 (0.6%) | 0.1897 | 0.637199 | ko05162 |
| 79 | Choline metabolism in cancer | 7 (1.37%) | 93 (0.93%) | 0.19533 | 0.647802 | ko05231 |
| 80 | Mannose type O-glycan biosynthesis | 2 (0.39%) | 17 (0.17%) | 0.213789 | 0.685058 | ko00515 |
| 81 | Legionellosis | 4 (0.78%) | 47 (0.47%) | 0.216552 | 0.685058 | ko05134 |
| 82 | Tryptophan metabolism | 4 (0.78%) | 47 (0.47%) | 0.216552 | 0.685058 | ko00380 |
| 83 | PPAR signaling pathway | 5 (0.98%) | 63 (0.63%) | 0.217022 | 0.685058 | ko03320 |
| 84 | Serotonergic synapse | 5 (0.98%) | 64 (0.64%) | 0.226391 | 0.706126 | ko04726 |
| 85 | Phenylalanine, tyrosine and tryptophan biosynthesis | 1 (0.2%) | 5 (0.05%) | 0.230172 | 0.707669 | ko00400 |
| 86 | Lysine degradation | 7 (1.37%) | 98 (0.98%) | 0.232288 | 0.707669 | ko00310 |
| 87 | Pathogenic Escherichia coli infection | 4 (0.78%) | 49 (0.49%) | 0.238617 | 0.718592 | ko05130 |
| 88 | Regulation of actin cytoskeleton | 8 (1.57%) | 117 (1.17%) | 0.246204 | 0.72236 | ko04810 |
| 89 | Hematopoietic cell lineage | 3 (0.59%) | 34 (0.34%) | 0.249342 | 0.72236 | ko04640 |
| 90 | Arachidonic acid metabolism | 3 (0.59%) | 34 (0.34%) | 0.249342 | 0.72236 | ko00590 |
| 91 | Transcriptional misregulation in cancer | 8 (1.57%) | 118 (1.18%) | 0.253385 | 0.72236 | ko05202 |
| 92 | Platinum drug resistance | 5 (0.98%) | 67 (0.67%) | 0.255168 | 0.72236 | ko01524 |
| 93 | RNA polymerase | 6 (1.17%) | 84 (0.84%) | 0.25641 | 0.72236 | ko03020 |
| 94 | Pentose phosphate pathway | 3 (0.59%) | 35 (0.35%) | 0.263285 | 0.728164 | ko00030 |
| 95 | Pancreatic secretion | 9 (1.76%) | 137 (1.37%) | 0.264029 | 0.728164 | ko04972 |
| 96 | Fanconi anemia pathway | 4 (0.78%) | 54 (0.54%) | 0.295701 | 0.803684 | ko03460 |
| 97 | Rap1 signaling pathway | 9 (1.76%) | 142 (1.42%) | 0.298695 | 0.803684 | ko04015 |
| 98 | ECM-receptor interaction | 6 (1.17%) | 89 (0.89%) | 0.300615 | 0.803684 | ko04512 |
| 99 | Melanogenesis | 5 (0.98%) | 72 (0.72%) | 0.304852 | 0.80678 | ko04916 |
| 100 | Glycosaminoglycan biosynthesis - heparan sulfate / heparin | 2 (0.39%) | 22 (0.22%) | 0.309843 | 0.811788 | ko00534 |
| 101 | Melanoma | 2 (0.39%) | 23 (0.23%) | 0.328915 | 0.849951 | ko05218 |
| 102 | Cysteine and methionine metabolism | 4 (0.78%) | 58 (0.58%) | 0.342493 | 0.849951 | ko00270 |
| 103 | Thyroid cancer | 2 (0.39%) | 24 (0.24%) | 0.347839 | 0.849951 | ko05216 |
| 104 | Fructose and mannose metabolism | 3 (0.59%) | 41 (0.41%) | 0.348125 | 0.849951 | ko00051 |
| 105 | Pyrimidine metabolism | 9 (1.76%) | 149 (1.49%) | 0.348887 | 0.849951 | ko00240 |
| 106 | Hippo signaling pathway | 7 (1.37%) | 113 (1.13%) | 0.353678 | 0.849951 | ko04390 |
| 107 | Leukocyte transendothelial migration | 4 (0.78%) | 59 (0.59%) | 0.354246 | 0.849951 | ko04670 |
| 108 | Hypertrophic cardiomyopathy (HCM) | 4 (0.78%) | 59 (0.59%) | 0.354246 | 0.849951 | ko05410 |
| 109 | Cell adhesion molecules (CAMs) | 6 (1.17%) | 95 (0.95%) | 0.355282 | 0.849951 | ko04514 |
| 110 | Ribosome biogenesis in eukaryotes | 7 (1.37%) | 114 (1.14%) | 0.36209 | 0.849951 | ko03008 |
| 111 | Arrhythmogenic right ventricular cardiomyopathy (ARVC) | 3 (0.59%) | 42 (0.42%) | 0.362268 | 0.849951 | ko05412 |
| 112 | Epstein-Barr virus infection | 12 (2.35%) | 207 (2.06%) | 0.365006 | 0.849951 | ko05169 |
| 113 | Leishmaniasis | 2 (0.39%) | 25 (0.25%) | 0.366582 | 0.849951 | ko05140 |
| 114 | Autoimmune thyroid disease | 1 (0.2%) | 9 (0.09%) | 0.375594 | 0.863207 | ko05320 |
| 115 | 2-Oxocarboxylic acid metabolism | 2 (0.39%) | 26 (0.26%) | 0.385112 | 0.874212 | ko01210 |
| 116 | Dilated cardiomyopathy (DCM) | 4 (0.78%) | 62 (0.62%) | 0.389464 | 0.874212 | ko05414 |
| 117 | Hedgehog signaling pathway | 3 (0.59%) | 44 (0.44%) | 0.390392 | 0.874212 | ko04340 |
| 118 | Small cell lung cancer | 5 (0.98%) | 81 (0.81%) | 0.396824 | 0.87457 | ko05222 |
| 119 | Cellular senescence | 6 (1.17%) | 100 (1%) | 0.401369 | 0.87457 | ko04218 |
| 120 | Base excision repair | 3 (0.59%) | 45 (0.45%) | 0.404343 | 0.87457 | ko03410 |
| 121 | FoxO signaling pathway | 5 (0.98%) | 82 (0.82%) | 0.407056 | 0.87457 | ko04068 |
| 122 | Glycosphingolipid biosynthesis - ganglio series | 1 (0.2%) | 10 (0.1%) | 0.407444 | 0.87457 | ko00604 |
| 123 | Glycerolipid metabolism | 6 (1.17%) | 101 (1.01%) | 0.410581 | 0.87457 | ko00561 |
| 124 | Bile secretion | 6 (1.17%) | 102 (1.02%) | 0.41978 | 0.886954 | ko04976 |
| 125 | HTLV-I infection | 10 (1.96%) | 179 (1.79%) | 0.429178 | 0.899557 | ko05166 |
| 126 | Selenocompound metabolism | 1 (0.2%) | 11 (0.11%) | 0.437672 | 0.902914 | ko00450 |
| 127 | Staphylococcus aureus infection | 1 (0.2%) | 11 (0.11%) | 0.437672 | 0.902914 | ko05150 |
| 128 | Pathways in cancer | 18 (3.52%) | 335 (3.34%) | 0.443139 | 0.90705 | ko05200 |
| 129 | Propanoate metabolism | 2 (0.39%) | 30 (0.3%) | 0.456613 | 0.914993 | ko00640 |
| 130 | Caffeine metabolism | 2 (0.39%) | 30 (0.3%) | 0.456613 | 0.914993 | ko00232 |
| 131 | Colorectal cancer | 3 (0.59%) | 49 (0.49%) | 0.459073 | 0.914993 | ko05210 |
| 132 | Tight junction | 8 (1.57%) | 145 (1.45%) | 0.460989 | 0.914993 | ko04530 |
| 133 | Prion diseases | 2 (0.39%) | 31 (0.31%) | 0.473737 | 0.929647 | ko05020 |
| 134 | Human papillomavirus infection | 13 (2.54%) | 244 (2.43%) | 0.475469 | 0.929647 | ko05165 |
| 135 | Cytosolic DNA-sensing pathway | 3 (0.59%) | 51 (0.51%) | 0.485632 | 0.935556 | ko04623 |
| 136 | Folate biosynthesis | 3 (0.59%) | 51 (0.51%) | 0.485632 | 0.935556 | ko00790 |
| 137 | Type I diabetes mellitus | 1 (0.2%) | 13 (0.13%) | 0.493589 | 0.937104 | ko04940 |
| 138 | Sulfur metabolism | 1 (0.2%) | 13 (0.13%) | 0.493589 | 0.937104 | ko00920 |
| 139 | Natural killer cell mediated cytotoxicity | 2 (0.39%) | 33 (0.33%) | 0.506983 | 0.948783 | ko04650 |
| 140 | Hedgehog signaling pathway - fly | 2 (0.39%) | 33 (0.33%) | 0.506983 | 0.948783 | ko04341 |
| 141 | Morphine addiction | 3 (0.59%) | 53 (0.53%) | 0.511548 | 0.949304 | ko05032 |
| 142 | Biosynthesis of amino acids | 4 (0.78%) | 73 (0.73%) | 0.514508 | 0.949304 | ko01230 |
| 143 | p53 signaling pathway | 4 (0.78%) | 75 (0.75%) | 0.536034 | 0.966944 | ko04115 |
| 144 | Insulin signaling pathway | 7 (1.37%) | 135 (1.35%) | 0.536617 | 0.966944 | ko04910 |
| 145 | Biosynthesis of unsaturated fatty acids | 2 (0.39%) | 35 (0.35%) | 0.538831 | 0.966944 | ko01040 |
| 146 | Amyotrophic lateral sclerosis (ALS) | 2 (0.39%) | 35 (0.35%) | 0.538831 | 0.966944 | ko05014 |
| 147 | Linoleic acid metabolism | 1 (0.2%) | 15 (0.15%) | 0.543956 | 0.9695 | ko00591 |
| 148 | Nitrogen metabolism | 1 (0.2%) | 16 (0.16%) | 0.567232 | 0.997703 | ko00910 |
| 149 | Estrogen signaling pathway | 4 (0.78%) | 78 (0.78%) | 0.567396 | 0.997703 | ko04915 |
| 150 | Homologous recombination | 2 (0.39%) | 38 (0.38%) | 0.583878 | 0.999545 | ko03440 |
| 151 | Focal adhesion | 8 (1.57%) | 162 (1.62%) | 0.587955 | 0.999545 | ko04510 |
| 152 | Amoebiasis | 4 (0.78%) | 81 (0.81%) | 0.597553 | 0.999545 | ko05146 |
| 153 | Nucleotide excision repair | 3 (0.59%) | 61 (0.61%) | 0.607698 | 0.999545 | ko03420 |
| 154 | Maturity onset diabetes of the young | 1 (0.2%) | 18 (0.18%) | 0.610287 | 0.999545 | ko04950 |
| 155 | Cell cycle | 5 (0.98%) | 104 (1.04%) | 0.617187 | 0.999545 | ko04110 |
| 156 | Pertussis | 2 (0.39%) | 41 (0.41%) | 0.625595 | 0.999545 | ko05133 |
| 157 | Viral carcinogenesis | 7 (1.37%) | 148 (1.48%) | 0.63465 | 0.999545 | ko05203 |
| 158 | DNA replication | 2 (0.39%) | 42 (0.42%) | 0.638761 | 0.999545 | ko03030 |
| 159 | Shigellosis | 3 (0.59%) | 64 (0.64%) | 0.640343 | 0.999545 | ko05131 |
| 160 | Cholinergic synapse | 4 (0.78%) | 86 (0.86%) | 0.644918 | 0.999545 | ko04725 |
| 161 | Thiamine metabolism | 1 (0.2%) | 20 (0.2%) | 0.649066 | 0.999545 | ko00730 |
| 162 | Bacterial invasion of epithelial cells | 3 (0.59%) | 66 (0.66%) | 0.661022 | 0.999545 | ko05100 |
| 163 | Renin secretion | 3 (0.59%) | 66 (0.66%) | 0.661022 | 0.999545 | ko04924 |
| 164 | Central carbon metabolism in cancer | 2 (0.39%) | 44 (0.44%) | 0.663998 | 0.999545 | ko05230 |
| 165 | MAPK signaling pathway | 8 (1.57%) | 175 (1.75%) | 0.674467 | 0.999545 | ko04010 |
| 166 | Amphetamine addiction | 3 (0.59%) | 68 (0.68%) | 0.680828 | 0.999545 | ko05031 |
| 167 | Toll and Imd signaling pathway | 5 (0.98%) | 112 (1.12%) | 0.681893 | 0.999545 | ko04624 |
| 168 | Dopaminergic synapse | 4 (0.78%) | 92 (0.92%) | 0.696706 | 0.999545 | ko04728 |
| 169 | Protein processing in endoplasmic reticulum | 8 (1.57%) | 179 (1.79%) | 0.698732 | 0.999545 | ko04141 |
| 170 | alpha-Linolenic acid metabolism | 1 (0.2%) | 23 (0.23%) | 0.700133 | 0.999545 | ko00592 |
| 171 | Ferroptosis | 1 (0.2%) | 23 (0.23%) | 0.700133 | 0.999545 | ko04216 |
| 172 | Hippo signaling pathway - fly | 4 (0.78%) | 93 (0.93%) | 0.70479 | 0.999545 | ko04391 |
| 173 | RNA degradation | 4 (0.78%) | 93 (0.93%) | 0.70479 | 0.999545 | ko03018 |
| 174 | B cell receptor signaling pathway | 2 (0.39%) | 48 (0.48%) | 0.710193 | 0.999545 | ko04662 |
| 175 | RNA transport | 9 (1.76%) | 203 (2.02%) | 0.713629 | 0.999545 | ko03013 |
| 176 | Glycerophospholipid metabolism | 5 (0.98%) | 117 (1.17%) | 0.718411 | 0.999545 | ko00564 |
| 177 | Pancreatic cancer | 2 (0.39%) | 49 (0.49%) | 0.720879 | 0.999545 | ko05212 |
| 178 | Fat digestion and absorption | 3 (0.59%) | 73 (0.73%) | 0.726546 | 0.999545 | ko04975 |
| 179 | Mismatch repair | 1 (0.2%) | 25 (0.25%) | 0.729986 | 0.999545 | ko03430 |
| 180 | Basal transcription factors | 2 (0.39%) | 50 (0.5%) | 0.731231 | 0.999545 | ko03022 |
| 181 | Sphingolipid metabolism | 2 (0.39%) | 50 (0.5%) | 0.731231 | 0.999545 | ko00600 |
| 182 | Olfactory transduction | 2 (0.39%) | 52 (0.52%) | 0.750959 | 0.999545 | ko04740 |
| 183 | Glyoxylate and dicarboxylate metabolism | 2 (0.39%) | 53 (0.53%) | 0.760348 | 0.999545 | ko00630 |
| 184 | Aldosterone synthesis and secretion | 3 (0.59%) | 78 (0.78%) | 0.76698 | 0.999545 | ko04925 |
| 185 | Non-small cell lung cancer | 1 (0.2%) | 28 (0.28%) | 0.769296 | 0.999545 | ko05223 |
| 186 | Jak-STAT signaling pathway | 1 (0.2%) | 28 (0.28%) | 0.769296 | 0.999545 | ko04630 |
| 187 | Axon guidance | 5 (0.98%) | 125 (1.25%) | 0.770453 | 0.999545 | ko04360 |
| 188 | Thyroid hormone synthesis | 2 (0.39%) | 55 (0.55%) | 0.778209 | 0.999545 | ko04918 |
| 189 | Fc epsilon RI signaling pathway | 1 (0.2%) | 29 (0.29%) | 0.781086 | 0.999545 | ko04664 |
| 190 | Ether lipid metabolism | 1 (0.2%) | 29 (0.29%) | 0.781086 | 0.999545 | ko00565 |
| 191 | Butanoate metabolism | 1 (0.2%) | 29 (0.29%) | 0.781086 | 0.999545 | ko00650 |
| 192 | MicroRNAs in cancer | 5 (0.98%) | 127 (1.27%) | 0.782252 | 0.999545 | ko05206 |
| 193 | PI3K-Akt signaling pathway | 8 (1.57%) | 195 (1.94%) | 0.783778 | 0.999545 | ko04151 |
| 194 | Tuberculosis | 4 (0.78%) | 105 (1.05%) | 0.789738 | 0.999545 | ko05152 |
| 195 | Viral myocarditis | 1 (0.2%) | 31 (0.31%) | 0.802893 | 0.999545 | ko05416 |
| 196 | Endocytosis | 7 (1.37%) | 178 (1.78%) | 0.809173 | 0.999545 | ko04144 |
| 197 | mTOR signaling pathway | 5 (0.98%) | 132 (1.32%) | 0.809697 | 0.999545 | ko04150 |
| 198 | Long-term depression | 1 (0.2%) | 32 (0.32%) | 0.812969 | 0.999545 | ko04730 |
| 199 | Calcium signaling pathway | 4 (0.78%) | 109 (1.09%) | 0.813304 | 0.999545 | ko04020 |
| 200 | Gap junction | 2 (0.39%) | 60 (0.6%) | 0.817843 | 0.999545 | ko04540 |
| 201 | Phospholipase D signaling pathway | 3 (0.59%) | 86 (0.86%) | 0.821426 | 0.999545 | ko04072 |
| 202 | Glycosylphosphatidylinositol (GPI)-anchor biosynthesis | 1 (0.2%) | 33 (0.33%) | 0.822531 | 0.999545 | ko00563 |
| 203 | Type II diabetes mellitus | 1 (0.2%) | 35 (0.35%) | 0.840216 | 0.999545 | ko04930 |
| 204 | NF-kappa B signaling pathway | 1 (0.2%) | 36 (0.36%) | 0.848388 | 0.999545 | ko04064 |
| 205 | Fatty acid elongation | 1 (0.2%) | 36 (0.36%) | 0.848388 | 0.999545 | ko00062 |
| 206 | Vasopressin-regulated water reabsorption | 1 (0.2%) | 37 (0.37%) | 0.856142 | 0.999545 | ko04962 |
| 207 | Prolactin signaling pathway | 1 (0.2%) | 37 (0.37%) | 0.856142 | 0.999545 | ko04917 |
| 208 | Taste transduction | 1 (0.2%) | 37 (0.37%) | 0.856142 | 0.999545 | ko04742 |
| 209 | Insulin secretion | 2 (0.39%) | 66 (0.66%) | 0.856956 | 0.999545 | ko04911 |
| 210 | Progesterone-mediated oocyte maturation | 2 (0.39%) | 67 (0.67%) | 0.862677 | 0.999545 | ko04914 |
| 211 | Endometrial cancer | 1 (0.2%) | 38 (0.38%) | 0.863501 | 0.999545 | ko05213 |
| 212 | HIF-1 signaling pathway | 2 (0.39%) | 68 (0.68%) | 0.868188 | 0.999545 | ko04066 |
| 213 | Notch signaling pathway | 1 (0.2%) | 41 (0.41%) | 0.883398 | 0.999545 | ko04330 |
| 214 | Alanine, aspartate and glutamate metabolism | 1 (0.2%) | 41 (0.41%) | 0.883398 | 0.999545 | ko00250 |
| 215 | Ovarian steroidogenesis | 1 (0.2%) | 41 (0.41%) | 0.883398 | 0.999545 | ko04913 |
| 216 | Apoptosis - fly | 2 (0.39%) | 74 (0.74%) | 0.897195 | 0.999545 | ko04214 |
| 217 | Th1 and Th2 cell differentiation | 1 (0.2%) | 44 (0.44%) | 0.900399 | 0.999545 | ko04658 |
| 218 | Glioma | 1 (0.2%) | 46 (0.46%) | 0.910335 | 0.999545 | ko05214 |
| 219 | Herpes simplex infection | 5 (0.98%) | 158 (1.58%) | 0.910857 | 0.999545 | ko05168 |
| 220 | Phototransduction - fly | 1 (0.2%) | 47 (0.47%) | 0.914926 | 0.999545 | ko04745 |
| 221 | Proteasome | 1 (0.2%) | 49 (0.49%) | 0.923415 | 0.999545 | ko03050 |
| 222 | Huntington's disease | 8 (1.57%) | 240 (2.39%) | 0.927837 | 0.999545 | ko05016 |
| 223 | Vibrio cholerae infection | 1 (0.2%) | 51 (0.51%) | 0.931059 | 0.999545 | ko05110 |
| 224 | Chronic myeloid leukemia | 1 (0.2%) | 51 (0.51%) | 0.931059 | 0.999545 | ko05220 |
| 225 | Dorso-ventral axis formation | 1 (0.2%) | 52 (0.52%) | 0.93459 | 0.999545 | ko04320 |
| 226 | Thyroid hormone signaling pathway | 3 (0.59%) | 115 (1.15%) | 0.937386 | 0.999545 | ko04919 |
| 227 | cGMP-PKG signaling pathway | 3 (0.59%) | 115 (1.15%) | 0.937386 | 0.999545 | ko04022 |
| 228 | Adherens junction | 2 (0.39%) | 86 (0.86%) | 0.938253 | 0.999545 | ko04520 |
| 229 | Salmonella infection | 1 (0.2%) | 56 (0.56%) | 0.947 | 0.999545 | ko05132 |
| 230 | AGE-RAGE signaling pathway in diabetic complications | 1 (0.2%) | 57 (0.57%) | 0.949716 | 0.999545 | ko04933 |
| 231 | T cell receptor signaling pathway | 1 (0.2%) | 58 (0.58%) | 0.952293 | 0.999545 | ko04660 |
| 232 | Spliceosome | 4 (0.78%) | 151 (1.51%) | 0.953313 | 0.999545 | ko03040 |
| 233 | Glutamatergic synapse | 1 (0.2%) | 59 (0.59%) | 0.954739 | 0.999545 | ko04724 |
| 234 | Gastric acid secretion | 1 (0.2%) | 59 (0.59%) | 0.954739 | 0.999545 | ko04971 |
| 235 | Fc gamma R-mediated phagocytosis | 1 (0.2%) | 60 (0.6%) | 0.957059 | 0.999545 | ko04666 |
| 236 | Longevity regulating pathway | 1 (0.2%) | 61 (0.61%) | 0.959261 | 0.999545 | ko04211 |
| 237 | Oocyte meiosis | 2 (0.39%) | 96 (0.96%) | 0.960059 | 0.999545 | ko04114 |
| 238 | Antifolate resistance | 1 (0.2%) | 65 (0.65%) | 0.966996 | 0.999545 | ko01523 |
| 239 | Inflammatory mediator regulation of TRP channels | 1 (0.2%) | 65 (0.65%) | 0.966996 | 0.999545 | ko04750 |
| 240 | Prostate cancer | 1 (0.2%) | 66 (0.66%) | 0.968689 | 0.999545 | ko05215 |
| 241 | NOD-like receptor signaling pathway | 2 (0.39%) | 103 (1.03%) | 0.970702 | 0.999545 | ko04621 |
| 242 | Endocrine resistance | 1 (0.2%) | 69 (0.69%) | 0.973265 | 0.999545 | ko01522 |
| 243 | Hepatitis C | 1 (0.2%) | 70 (0.7%) | 0.974637 | 0.999545 | ko05160 |
| 244 | Hepatitis B | 1 (0.2%) | 70 (0.7%) | 0.974637 | 0.999545 | ko05161 |
| 245 | Glucagon signaling pathway | 1 (0.2%) | 73 (0.73%) | 0.978346 | 0.999545 | ko04922 |
| 246 | Epithelial cell signaling in Helicobacter pylori infection | 1 (0.2%) | 75 (0.75%) | 0.980512 | 0.999545 | ko05120 |
| 247 | Necroptosis | 1 (0.2%) | 78 (0.78%) | 0.983363 | 0.999545 | ko04217 |
| 248 | cAMP signaling pathway | 5 (0.98%) | 209 (2.08%) | 0.98384 | 0.999545 | ko04024 |
| 249 | Relaxin signaling pathway | 1 (0.2%) | 79 (0.79%) | 0.984217 | 0.999545 | ko04926 |
| 250 | Salivary secretion | 1 (0.2%) | 80 (0.8%) | 0.985028 | 0.999545 | ko04970 |
| 251 | Ras signaling pathway | 3 (0.59%) | 155 (1.55%) | 0.987308 | 0.999545 | ko04014 |
| 252 | Ubiquitin mediated proteolysis | 3 (0.59%) | 159 (1.59%) | 0.989256 | 0.999545 | ko04120 |
| 253 | Retrograde endocannabinoid signaling | 1 (0.2%) | 90 (0.9%) | 0.991166 | 0.999545 | ko04723 |
| 254 | Oxytocin signaling pathway | 1 (0.2%) | 92 (0.92%) | 0.992052 | 0.999545 | ko04921 |
| 255 | Chemokine signaling pathway | 1 (0.2%) | 94 (0.94%) | 0.992848 | 0.999545 | ko04062 |
| 256 | Parkinson's disease | 2 (0.39%) | 135 (1.35%) | 0.993179 | 0.999545 | ko05012 |
| 257 | Insulin resistance | 1 (0.2%) | 100 (1%) | 0.994791 | 0.999545 | ko04931 |
| 258 | MAPK signaling pathway - fly | 2 (0.39%) | 156 (1.56%) | 0.997452 | 0.999545 | ko04013 |
| 259 | Alzheimer's disease | 2 (0.39%) | 170 (1.7%) | 0.998691 | 0.999545 | ko05010 |
| 260 | Non-alcoholic fatty liver disease (NAFLD) | 1 (0.2%) | 128 (1.28%) | 0.998816 | 0.999545 | ko04932 |
| 261 | Oxidative phosphorylation | 1 (0.2%) | 129 (1.29%) | 0.998878 | 0.999545 | ko00190 |
| 262 | Ribosome | 1 (0.2%) | 146 (1.46%) | 0.999545 | 0.999545 | ko03010 |

Note: Pathways with *Q*-value ≤ 0.05 are significantly enriched pathways in DEGs comparing with the whole genome background.
