# Supplementary material for: Insecticidal Activity of Artemisia vulgaris Essential Oil and Transcriptome Analysis of Tribolium castaneum in Response to Oil Exposure
Source: Front Genet. 2020 Jun 25;11:589. doi: 10.3389/fgene.2020.00589 (PMC7330086; doi:10.3389/fgene.2020.00589)
Supplement: TABLE S3 — A set of 758 differentially expressed genes (DEGs) between the 5% Artemisia vulgaris treatment and control groups. [file Table_3.docx]

**Supplementary Table S3** A set of 758 DEGs between the 5% *A. vulgaris* treatment and control groups

| Gene ID | Log_2_Ratio (T/C) | Regulation (T/C) | *P*-value | Protein |
| --- | --- | --- | --- | --- |
| LOC103313288 | 9.06 | Up | 2.23E-51 | N-acetyltransferase |
| LOC658008 | 8.97 | Up | 6.44E-49 | Mitochondrial ribosomal protein L16-like protein |
| LOC100142044 | 8.17 | Up | 2.17E-31 | Misexpression suppressor of ras 4 |
| LOC103315142 | 7.95 | Up | 7.82E-28 | / |
| LOC107398862 | 7.14 | Up | 1.66E-17 | / |
| LOC659416 | 7.12 | Up | 2.53E-17 | Activated CDC42 kinase 1 |
| LOC655078 | 6.97 | Up | 6.03E-16 | / |
| LOC103312786 | 6.64 | Up | 3.32E-13 | / |
| LOC103312788 | 6.09 | Up | 2.22E-226 | Cuticle protein 65-like |
| LOC655017 | 5.98 | Up | 5.15E-09 | Moesin/ezrin/radixin homolog 1 |
| LOC103312802 | 5.98 | Up | 1.54E-16 | Serine/arginine repetitive matrix protein 4 |
| LOC654921 | 5.91 | Up | 0 | Cuticle protein 64 |
| LOC654983 | 5.62 | Up | 0 | Cuticle protein 63 |
| LOC656963 | 5.48 | Up | 9.37E-07 | WD repeat-containing protein mio-B-like |
| LOC103314651 | 4.91 | Up | 6.90E-05 | / |
| LOC107398018 | 4.72 | Up | 0.0002051 | / |
| LOC107398985 | 4.62 | Up | 0.0003693 | / |
| LOC660329 | 4.47 | Up | 2.17E-06 | Microtubule-associated protein 1B |
| LOC107398214 | 4.46 | Up | 0.0008173 | Titin-like |
| LOC103313073 | 4.44 | Up | 3.24E-79 | Major royal jelly protein 3 |
| LOC107399017 | 4.42 | Up | 0.0010064 | RNA-directed DNA polymerase from mobile element jockey-like |
| LOC656161 | 4.28 | Up | 2.26E-65 | OBPC11 |
| LOC656347 | 4.21 | Up | 1.07E-25 | Cuticle protein 7 |
| LOC658485 | 4.17 | Up | 2.56E-05 | Carbonic anhydrase 1 |
| LOC660270 | 4.03 | Up | 0 | CYP4BN1 |
| LOC103312637 | 4.00 | Up | 2.95E-08 | / |
| LOC655437 | 3.83 | Up | 3.87E-16 | Cuticle protein 64 |
| LOC103314218 | 3.81 | Up | 0.0003071 | / |
| LOC103313692 | 3.79 | Up | 4.77E-41 | Adfb like protein |
| LOC655196 | 3.78 | Up | 2.10E-29 | Cuticle protein 63 |
| LOC660050 | 3.73 | Up | 0.0003508 | Carboxypeptidase A |
| LOC103314344 | 3.70 | Up | 0.0005743 | Retinol dehydrogenase 14 |
| LOC103312304 | 3.70 | Up | 0.0005743 | Peptide transporter family 1 |
| LOC660054 | 3.70 | Up | 0.0005743 | Testis-specific serine/threonine-protein kinase 3 |
| LOC103313963 | 3.67 | Up | 3.38E-105 | / |
| LOC660166 | 3.62 | Up | 1.75E-23 | Protein G12-like |
| LOC657675 | 3.58 | Up | 0.0010758 | Osiris19 |
| LOC658313 | 3.58 | Up | 0.0010758 | Sperm flagellar protein 1 |
| LOC103312783 | 3.58 | Up | 0.0010826 | Crisp/allergen/pr-1 |
| LOC103314908 | 3.58 | Up | 4.53E-27 | / |
| LOC664115 | 3.56 | Up | 3.46E-16 | Probable serine/threonine-protein kinase tsua |
| LOC103314113 | 3.54 | Up | 0.0012732 | / |
| LOC100142122 | 3.46 | Up | 2.72E-07 | Histone H1B |
| LOC103313150 | 3.46 | Up | 1.26E-05 | Venom allergen 5-like |
| LOC103313123 | 3.43 | Up | 7.55E-06 | / |
| LOC657788 | 3.15 | Up | 4.91E-65 | Cuticle protein 38 |
| LOC656922 | 3.14 | Up | 2.33E-32 | Keratin-associated protein 10-8 |
| LOC103313766 | 3.12 | Up | 0 | Cuticle protein |
| LOC103314159 | 3.09 | Up | 0.0002586 | Glutamine-rich protein 2-like |
| LOC100142317 | 3.08 | Up | 0 | HSP68a |
| LOC103312598 | 3.03 | Up | 1.24E-61 | Major royal jelly protein 3 |
| LOC659269 | 3.00 | Up | 0.0004711 | / |
| LOC662640 | 2.99 | Up | 1.77E-21 | Facilitated trehalose transporter Tret1 |
| LOC658343 | 2.92 | Up | 1.06E-24 | CP 1 |
| LOC654890 | 2.91 | Up | 5.11E-16 | Condensin complex subunit 2 |
| LOC100142517 | 2.87 | Up | 0 | HSP68b |
| LOC655601 | 2.80 | Up | 0 | Adult-specific cuticular protein ACP-20-like Protein |
| LOC657612 | 2.75 | Up | 0 | / |
| LOC664358 | 2.74 | Up | 0.0001893 | / |
| LOC107398123 | 2.72 | Up | 1.74E-06 | Neurofilament heavy polypeptide-like |
| LOC660181 | 2.71 | Up | 4.09E-59 | Beta-1,4-glucuronyltransferase 1 |
| LOC107399181 | 2.71 | Up | 4.30E-09 | Major royal jelly protein 3-like |
| LOC662967 | 2.70 | Up | 2.38E-05 | IQ and AAA domain-containing protein 1-like |
| LOC107399133 | 2.68 | Up | 0 | ORF7 |
| LOC655167 | 2.68 | Up | 0 | Protein takeout-like |
| LOC103313803 | 2.67 | Up | 6.01E-42 | Major royal jelly protein 3 |
| LOC103314866 | 2.64 | Up | 0 | Probable WRKY transcription factor protein 1 |
| LOC107398973 | 2.63 | Up | 2.77E-44 | Gametocyte-specific factor 1 homolog |
| LOC660669 | 2.62 | Up | 8.30E-75 | Cat L precursor |
| LOC103314422 | 2.58 | Up | 7.34E-05 | Cuticular protein 76Bb |
| LOC660306 | 2.58 | Up | 7.80E-05 | DPY30 domain-containing protein 1 |
| LOC103314549 | 2.52 | Up | 0.0001282 | Nose resistant to fluoxetine protein 6-like |
| LOC103314867 | 2.52 | Up | 2.20E-122 | Zinc metalloproteinase nas-4 |
| LOC100142614 | 2.51 | Up | 7.72E-75 | Keratin-associated protein 19-9b |
| LOC103314477 | 2.50 | Up | 2.11E-09 | / |
| LOC664422 | 2.50 | Up | 0 | Gadd45 |
| LOC664159 | 2.49 | Up | 3.17E-81 | Enolase |
| LOC661258 | 2.48 | Up | 6.29E-284 | Adult cuticle protein 1-like |
| LOC658925 | 2.45 | Up | 0 | / |
| LOC107398532 | 2.43 | Up | 1.09E-36 | / |
| LOC103313602 | 2.43 | Up | 7.04E-210 | / |
| LOC103313332 | 2.43 | Up | 5.64E-81 | / |
| LOC107398518 | 2.41 | Up | 5.30E-06 | / |
| LOC100313959 | 2.41 | Up | 0 | Peritrophic matrix protein 14 |
| LOC100142058 | 2.40 | Up | 0 | Keratin, type I cytoskeletal 9 |
| LOC662168 | 2.38 | Up | 1.11E-202 | HSP27 |
| LOC658137 | 2.34 | Up | 1.13E-211 | Brachyurin |
| LOC107398068 | 2.32 | Up | 3.08E-05 | / |
| LOC103313694 | 2.31 | Up | 2.42E-22 | Adfb like protein |
| LOC103312756 | 2.31 | Up | 1.42E-13 | Cubilin-like Protein |
| LOC103314997 | 2.29 | Up | 1.47E-119 | Ankyrin repeat protein |
| LOC655042 | 2.29 | Up | 5.59E-07 | Glucose dehydrogenase |
| LOC662285 | 2.24 | Up | 0 | Larval/pupal cuticle protein H1C |
| LOC660732 | 2.20 | Up | 5.10E-149 | / |
| LOC662935 | 2.19 | Up | 3.23E-05 | Spermine oxidase |
| LOC100142515 | 2.18 | Up | 4.53E-95 | Calpain-A |
| LOC103314473 | 2.12 | Up | 9.25E-12 | / |
| LOC100240681 | 2.12 | Up | 6.93E-09 | OBPC17 |
| LOC103313863 | 2.12 | Up | 7.22E-06 | / |
| LOC663364 | 2.10 | Up | 5.37E-218 | DETS 6 |
| LOC103314140 | 2.10 | Up | 7.56E-19 | / |
| LOC107398732 | 2.08 | Up | 3.14E-07 | Nose resistant to fluoxetine protein 6-like |
| LOC103313025 | 2.08 | Up | 1.19E-05 | / |
| LOC659367 | 2.08 | Up | 1.84E-77 | Cat L precursor |
| LOC662363 | 2.05 | Up | 0.0001489 | ADAMTS7 |
| LOC664471 | 2.04 | Up | 5.36E-52 | CYP6BQ7 |
| LOC103314384 | 2.04 | Up | 4.19E-09 | / |
| LOC664569 | 2.04 | Up | 0 | Beta-hexosaminidase subunit alpha |
| LOC103315022 | 2.04 | Up | 2.18E-10 | Phospholipase A2 inhibitor |
| LOC658048 | 2.03 | Up | 2.33E-37 | CYP6A2 |
| LOC655587 | 2.02 | Up | 2.13E-51 | Peroxidase |
| LOC661267 | 2.01 | Up | 4.83E-226 | Glucose dehydrogenase |
| LOC663216 | 2.00 | Up | 7.51E-88 | Alpha-crystallin A chain |
| LOC663215 | 1.96 | Up | 3.22E-17 | MFS15 |
| LOC657293 | 1.96 | Up | 0 | Serine protease H107 |
| LOC103312691 | 1.94 | Up | 1.25E-20 | / |
| LOC660970 | 1.91 | Up | 0 | Proline-rich extensin-like protein EPR1 |
| LOC662126 | 1.91 | Up | 1.89E-10 | Glucose dehydrogenase-like |
| LOC107398166 | 1.91 | Up | 1.98E-07 | Ugt86Dg |
| LOC664595 | 1.91 | Up | 2.23E-217 | OBP10 |
| LOC100142357 | 1.90 | Up | 2.57E-06 | Spermine oxidase-like |
| LOC659076 | 1.89 | Up | 9.65E-09 | / |
| LOC662506 | 1.87 | Up | 0 | α-EST5 |
| LOC660127 | 1.87 | Up | 0 | / |
| LOC662964 | 1.86 | Up | 0 | Endocuticle structural glycoprotein sgabd-2 |
| LOC658401 | 1.86 | Up | 0 | Beta-glucosidase |
| LOC657893 | 1.85 | Up | 1.10E-07 | / |
| LOC657754 | 1.85 | Up | 0 | Serine protease H105 |
| LOC659203 | 1.84 | Up | 3.68E-06 | ATP-binding cassette sub-family A member 3 |
| LOC661071 | 1.82 | Up | 9.98E-06 | / |
| LOC100141891 | 1.82 | Up | 0 | Glycine-rich RNA-binding protein blt801 |
| LOC655464 | 1.82 | Up | 7.91E-92 | Osiris-like protein |
| LOC662996 | 1.81 | Up | 0 | Peritrophic matrix protein 1-C |
| LOC655486 | 1.81 | Up | 4.92E-137 | ABC transporter A family member 1 |
| LOC657117 | 1.81 | Up | 1.26E-06 | Cat B1 liike |
| LOC107398158 | 1.79 | Up | 7.41E-05 | Cat L1 like |
| LOC103314348 | 1.78 | Up | 6.05E-10 | Golgin subfamily A member 5-like |
| LOC662274 | 1.78 | Up | 1.70E-94 | Deoxycytidylate deaminase |
| LOC655388 | 1.78 | Up | 1.06E-06 | Osiris9 |
| LOC658340 | 1.78 | Up | 0 | UNC93-like protein MFSD11 |
| LOC107398511 | 1.78 | Up | 1.05E-11 | Aldose reductase-like |
| LOC657602 | 1.76 | Up | 3.47E-76 | GSTs6 |
| LOC100141698 | 1.76 | Up | 2.86E-74 | Yellow-e |
| LOC103314173 | 1.76 | Up | 6.29E-109 | Ankyrin-3-like |
| LOC103313347 | 1.76 | Up | 7.04E-37 | Mucin-22-like |
| LOC100142048 | 1.76 | Up | 8.09E-101 | Atlastin |
| LOC107397828 | 1.75 | Up | 0.0009787 | Odorant receptor Or1-like |
| LOC659538 | 1.75 | Up | 6.64E-09 | Protamine |
| LOC657696 | 1.74 | Up | 3.34E-45 | Skin secretory protein xp2-like |
| LOC107399202 | 1.74 | Up | 1.74E-11 | / |
| LOC103314594 | 1.74 | Up | 6.78E-05 | Pancreatic lipase-related protein 2 |
| LOC662970 | 1.73 | Up | 0 | Peritrophic matrix protein 3 |
| LOC662543 | 1.73 | Up | 0 | Inter-alpha-trypsin inhibitor heavy chain H4 |
| LOC103313754 | 1.71 | Up | 1.66E-06 | Cuticle protein 65 |
| LOC657476 | 1.69 | Up | 1.42E-18 | / |
| LOC663050 | 1.68 | Up | 5.13E-23 | / |
| LOC662478 | 1.68 | Up | 3.52E-12 | Endocuticle structural glycoprotein sgabd-8-like |
| LOC103314109 | 1.67 | Up | 0.0006522 | / |
| LOC655053 | 1.67 | Up | 1.56E-11 | Male sterile (3) 76Ca |
| LOC658517 | 1.67 | Up | 3.44E-10 | Tubulin beta chain |
| LOC664138 | 1.66 | Up | 8.26E-20 | Protein lin-28 homolog |
| LOC659885 | 1.66 | Up | 1.39E-50 | Serine protease P123 |
| LOC662442 | 1.66 | Up | 0 | Inter-alpha-trypsin inhibitor heavy chain H4 |
| LOC655395 | 1.65 | Up | 9.60E-05 | Fatty acyl-coa reductase 1 |
| LOC103314755 | 1.65 | Up | 1.43E-33 | Ankyrin-1-like |
| LOC103313645 | 1.65 | Up | 5.77E-13 | / |
| LOC107398348 | 1.64 | Up | 1.55E-07 | / |
| LOC103313594 | 1.64 | Up | 3.07E-10 | Cytochrome b561 domain-containing protein 2-like |
| LOC103313256 | 1.63 | Up | 7.01E-07 | Neuropeptide-like protein 31 |
| LOC659754 | 1.63 | Up | 5.42E-34 | Beta-1,4-glucuronyltransferase 1 |
| LOC103313315 | 1.63 | Up | 6.44E-23 | CYP351A2 |
| LOC107398771 | 1.62 | Up | 8.49E-11 | / |
| LOC663240 | 1.62 | Up | 7.82E-181 | Protein lethal(2) essential for life |
| LOC663523 | 1.62 | Up | 1.87E-19 | Cytosol aminopeptidase |
| LOC660410 | 1.62 | Up | 5.48E-09 | Protein MLP1 homolog |
| LOC662658 | 1.59 | Up | 0 | 4-hydroxyphenylpyruvate dioxygenase |
| LOC103312106 | 1.58 | Up | 2.96E-27 | / |
| LOC100141953 | 1.58 | Up | 2.82E-06 | Ugt2B7-like |
| LOC100141722 | 1.58 | Up | 7.54E-05 | Octopamine receptor 1 |
| LOC657133 | 1.58 | Up | 6.93E-110 | Serine protease H115 |
| LOC661736 | 1.57 | Up | 9.00E-07 | Centrosomal protein of 131 kda |
| LOC103312160 | 1.57 | Up | 1.15E-18 | / |
| LOC103314193 | 1.56 | Up | 2.00E-09 | Clavesin-2-like |
| LOC660891 | 1.55 | Up | 8.47E-20 | Myrosinase 1-like |
| LOC103315172 | 1.54 | Up | 0.0010768 | Larval cuticle protein A2B-like |
| LOC659137 | 1.54 | Up | 1.13E-103 | Solute carrier family 22 member 1 |
| LOC103314306 | 1.53 | Up | 7.46E-28 | Tyrosine recombinase, partial |
| LOC662695 | 1.53 | Up | 4.98E-09 | Camp and camp-inhibited cgmp 3',5'-cyclic phosphodiesterase 10A |
| LOC103313693 | 1.53 | Up | 2.37E-34 | Cuticle protein 19.8-like |
| LOC657270 | 1.53 | Up | 8.08E-40 | Farnesol dehydrogenase |
| LOC663932 | 1.53 | Up | 3.16E-134 | Venom dipeptidyl peptidase 4 |
| LOC107398924 | 1.52 | Up | 2.22E-248 | HTH-type transcriptional regulator hdfr |
| LOC103313584 | 1.52 | Up | 0.0001756 | / |
| LOC662886 | 1.51 | Up | 5.75E-87 | Sialin |
| LOC103314776 | 1.51 | Up | 0 | / |
| LOC103313789 | 1.51 | Up | 2.22E-47 | / |
| LOC103314564 | 1.51 | Up | 3.17E-55 | TBC1 domain family member 30 |
| LOC658944 | 1.51 | Up | 0 | Fatty acid synthase |
| LOC103313695 | 1.51 | Up | 5.07E-61 | Cuticle protein 16.5, isoform A-like |
| LOC658927 | 1.51 | Up | 0.000886 | Pupal cuticle protein C1B |
| LOC664243 | 1.50 | Up | 7.05E-25 | Aldose reductase |
| LOC655384 | 1.50 | Up | 0.0009372 | Amine sulfotransferase |
| LOC103312761 | 1.50 | Up | 5.06E-24 | Lamin-B receptor |
| LOC661563 | 1.48 | Up | 2.63E-245 | Lysosomal Pro-X carboxypeptidase |
| LOC663293 | 1.48 | Up | 0 | HSP70a |
| LOC100141821 | 1.48 | Up | 4.30E-12 | Snmp1 |
| LOC103313691 | 1.47 | Up | 2.04E-10 | / |
| LOC660851 | 1.47 | Up | 1.13E-08 | 15-hydroxyprostaglandin dehydrogenase [NAD(+)]-like |
| LOC656937 | 1.47 | Up | 1.65E-114 | MRP4 |
| LOC657146 | 1.46 | Up | 2.25E-61 | / |
| LOC660916 | 1.45 | Up | 0 | Proline-rich extensin-like protein EPR1 |
| LOC100142066 | 1.44 | Up | 0 | Protein slit-like Protein |
| LOC662755 | 1.44 | Up | 4.77E-57 | Toll-like receptor 2 |
| LOC107398155 | 1.43 | Up | 7.72E-164 | Carboxypeptidase N subunit 2-like |
| LOC661102 | 1.43 | Up | 1.12E-09 | α-EST2 |
| LOC103314504 | 1.43 | Up | 1.30E-38 | Choline transporter-like protein 1 |
| LOC107397756 | 1.42 | Up | 4.86E-49 | / |
| LOC662714 | 1.42 | Up | 2.62E-27 | / |
| LOC103314168 | 1.42 | Up | 1.08E-12 | / |
| LOC661167 | 1.42 | Up | 2.13E-67 | Intraflagellar transport protein 56 |
| LOC663474 | 1.42 | Up | 6.39E-66 | Protein takeout |
| LOC657594 | 1.41 | Up | 3.34E-20 | Equilibrative nucleoside transporter 3-like |
| LOC103312677 | 1.41 | Up | 3.04E-156 | Mucin 14a |
| LOC664599 | 1.41 | Up | 5.71E-155 | OBPC02 |
| LOC659039 | 1.41 | Up | 3.36E-28 | Lipase member K |
| LOC656243 | 1.40 | Up | 0 | OBPC12 |
| LOC103314642 | 1.40 | Up | 3.57E-06 | / |
| LOC103313746 | 1.40 | Up | 0 | Vanin-like protein 1 |
| LOC661111 | 1.40 | Up | 0 | Myrosinase 1-like |
| LOC103314393 | 1.40 | Up | 0.000494 | / |
| LOC100142345 | 1.39 | Up | 2.24E-80 | Putative uncharacterized protein ART2 |
| LOC656451 | 1.39 | Up | 2.10E-15 | HSP70b |
| LOC659485 | 1.38 | Up | 9.39E-137 | ATP-binding cassette sub-family A member 3-like |
| LOC103313304 | 1.38 | Up | 9.21E-12 | Chymotrypsin-1 |
| LOC658557 | 1.38 | Up | 1.56E-11 | Cpr47Ef |
| LOC661621 | 1.37 | Up | 0 | α-EST6 |
| LOC660957 | 1.37 | Up | 0 | CuZnSOD |
| LOC662962 | 1.37 | Up | 2.70E-101 | Dopa decarboxylase |
| LOC103313351 | 1.37 | Up | 4.53E-23 | Ankyrin 2 |
| LOC103313893 | 1.37 | Up | 2.33E-26 | Twik family of potassium channels protein 7 |
| LOC103314939 | 1.37 | Up | 7.94E-35 | Transcription factor GATA-4 |
| LOC663642 | 1.37 | Up | 0 | / |
| LOC659321 | 1.36 | Up | 0 | Tyrosine aminotransferase |
| LOC103313770 | 1.36 | Up | 1.44E-15 | / |
| LOC661270 | 1.36 | Up | 2.72E-31 | CSP12 |
| LOC100141982 | 1.35 | Up | 2.13E-139 | Methyltransferase 1 |
| LOC100141587 | 1.35 | Up | 9.52E-73 | Trypsin alpha-3 |
| LOC103314465 | 1.35 | Up | 6.43E-40 | Leucine-rich repeat-containing protein let-4 |
| LOC654861 | 1.35 | Up | 1.60E-84 | Iodotyrosine deiodinase 1 |
| LOC661428 | 1.35 | Up | 0 | Chitinase 8 |
| LOC103313485 | 1.35 | Up | 1.23E-11 | / |
| LOC658110 | 1.35 | Up | 0 | Cuticle protein 16.5, isoform B |
| LOC657363 | 1.35 | Up | 1.39E-86 | Serine-pyruvate aminotransferase |
| LOC659494 | 1.34 | Up | 1.10E-65 | Farnesol dehydrogenase |
| LOC103314519 | 1.34 | Up | 3.15E-140 | Branchless |
| LOC662321 | 1.34 | Up | 0 | Adfb like protein |
| LOC657195 | 1.34 | Up | 6.57E-278 | Ecdysone-inducible gene L2 |
| LOC100142258 | 1.34 | Up | 8.59E-67 | Serine/threonine-protein phosphatase 6 regulatory ankyrin repeat subunit B-like isoform X2 |
| LOC657697 | 1.33 | Up | 0 | CSP20 |
| LOC659290 | 1.33 | Up | 1.31E-242 | Cytochrome b5-related protein |
| LOC103312712 | 1.33 | Up | 1.51E-14 | / |
| LOC656589 | 1.33 | Up | 0 | L-lactate dehydrogenase |
| LOC663797 | 1.33 | Up | 1.71E-123 | Juvenile hormone-inducible protein |
| LOC103315021 | 1.32 | Up | 4.46E-29 | Slit homolog 1 protein-like |
| LOC660187 | 1.32 | Up | 3.46E-108 | Putative fatty acyl-coa reductase |
| LOC664285 | 1.31 | Up | 2.63E-37 | CYP9Z2 |
| LOC103312152 | 1.31 | Up | 4.10E-252 | Glutamyl aminopeptidase-like |
| LOC661518 | 1.31 | Up | 1.83E-45 | Dumpy |
| LOC100141808 | 1.30 | Up | 4.08E-08 | Sina homologue |
| LOC103312286 | 1.30 | Up | 5.53E-295 | Lipopolysaccharide-induced tumor necrosis factor-alpha factor homolog |
| LOC103312818 | 1.30 | Up | 0.000999 | Ras-specific guanine nucleotide-releasing factor ralgps1 |
| LOC103313968 | 1.29 | Up | 6.35E-55 | / |
| LOC655704 | 1.29 | Up | 0.0009889 | Dynein heavy chain 2, axonemal |
| LOC661831 | 1.29 | Up | 0 | Aminopeptidase N-like protein |
| LOC100141988 | 1.29 | Up | 4.27E-06 | Zinc finger protein with KRAB and SCAN domains 1 |
| LOC103312383 | 1.28 | Up | 3.62E-21 | Chymotrypsin-1 |
| LOC660377 | 1.28 | Up | 0 | Putative protein TPRXL isoform X2 |
| LOC664564 | 1.28 | Up | 3.89E-07 | Glucosylceramidase-like |
| LOC657454 | 1.27 | Up | 2.98E-30 | CYP9AC1 |
| LOC103313617 | 1.27 | Up | 3.15E-17 | / |
| LOC103312611 | 1.27 | Up | 5.97E-38 | / |
| LOC103313165 | 1.27 | Up | 1.61E-46 | Venom protease |
| LOC103312694 | 1.27 | Up | 1.31E-08 | / |
| LOC103313283 | 1.27 | Up | 1.23E-05 | / |
| LOC660551 | 1.26 | Up | 1.04E-307 | Cat L |
| LOC103314502 | 1.25 | Up | 0.000482 | Choline transporter-like protein 1 |
| LOC659584 | 1.25 | Up | 8.45E-74 | Serine protease H143 |
| LOC103314202 | 1.25 | Up | 2.13E-94 | / |
| LOC662874 | 1.24 | Up | 1.34E-139 | Myosin 7B |
| LOC661469 | 1.24 | Up | 0.000982 | CSP17 |
| LOC103314500 | 1.24 | Up | 1.79E-12 | 15-hydroxyprostaglandin dehydrogenase [NAD(+)]-like |
| LOC662610 | 1.24 | Up | 3.52E-90 | Putative fatty acyl-coa reductase CG5065 |
| LOC656030 | 1.24 | Up | 0.000406 | Titin |
| LOC661842 | 1.24 | Up | 1.04E-07 | Aromatic-L-amino-acid decarboxylase |
| LOC659255 | 1.24 | Up | 0.000169 | Protein phosphatase 1c interacting |
| LOC103312165 | 1.23 | Up | 0.0001424 | Odorant receptor 4 |
| LOC659571 | 1.23 | Up | 3.20E-08 | / |
| LOC107398213 | 1.22 | Up | 2.12E-05 | Transient receptor potential cation channel protein painless-like |
| LOC656120 | 1.22 | Up | 1.19E-27 | Ugt2B16 |
| LOC103313383 | 1.22 | Up | 2.25E-22 | / |
| LOC656897 | 1.21 | Up | 0 | / |
| LOC103312559 | 1.21 | Up | 6.37E-08 | / |
| LOC663447 | 1.21 | Up | 1.74E-109 | Fatty-acid amide hydrolase 2-B |
| LOC662865 | 1.20 | Up | 7.15E-05 | Acyl-coa desaturase 1 |
| LOC107398453 | 1.20 | Up | 0.0001709 | Polycystic kidney disease protein 1-like 2 |
| LOC662614 | 1.19 | Up | 3.16E-06 | Spermine oxidase |
| LOC658235 | 1.19 | Up | 3.30E-75 | Serine protease H122 |
| LOC107398361 | 1.19 | Up | 1.24E-60 | Probable cyclin-dependent serine/threonine-protein kinase |
| LOC659138 | 1.18 | Up | 0 | Bifunctional 3'-phosphoadenosine 5'-phosphosulfate synthase |
| LOC103313391 | 1.18 | Up | 3.23E-22 | / |
| LOC658199 | 1.18 | Up | 0 | Protein mesh |
| LOC660404 | 1.17 | Up | 1.69E-45 | / |
| LOC103314560 | 1.17 | Up | 3.99E-41 | Methuselah |
| LOC659881 | 1.16 | Up | 6.00E-05 | Regulating synaptic membrane exocytosis protein 2 |
| LOC107399201 | 1.16 | Up | 2.27E-36 | / |
| LOC663629 | 1.16 | Up | 0.0005907 | Larval/pupal cuticle protein H1C |
| LOC103313392 | 1.16 | Up | 2.73E-08 | Collagenase |
| LOC660229 | 1.15 | Up | 2.28E-143 | / |
| LOC661414 | 1.15 | Up | 1.63E-08 | Cytosol aminopeptidase |
| LOC659207 | 1.15 | Up | 4.57E-54 | Transferrin |
| LOC103313132 | 1.15 | Up | 9.87E-83 | Pastrel, isoform F |
| LOC100142186 | 1.15 | Up | 1.74E-09 | Nose resistant to fluoxetine protein 6-like |
| LOC659847 | 1.15 | Up | 4.96E-63 | MRP49 |
| LOC100141575 | 1.15 | Up | 4.45E-08 | Brachyurin-like |
| LOC655370 | 1.14 | Up | 8.74E-37 | Tequila |
| LOC660391 | 1.14 | Up | 0 | Basic proline-rich protein |
| LOC655834 | 1.14 | Up | 5.26E-212 | Ancylostoma secreted protein-like isoform X5 |
| LOC659009 | 1.13 | Up | 4.50E-45 | GSTs7 |
| LOC659020 | 1.13 | Up | 4.76E-132 | / |
| LOC663183 | 1.13 | Up | 9.94E-51 | Juvenile hormone-inducible protein 26 |
| LOC103313341 | 1.13 | Up | 4.32E-11 | / |
| LOC656770 | 1.12 | Up | 6.93E-55 | CYP6BK11 |
| LOC661219 | 1.12 | Up | 1.22E-37 | CSP11 |
| LOC661658 | 1.12 | Up | 1.90E-40 | Cuticle protein 16.5, isoform A |
| LOC657694 | 1.12 | Up | 0 | Hexokinase type 2 |
| LOC662588 | 1.12 | Up | 2.65E-130 | Sodium-dependent nutrient amino acid transporter 1 |
| LOC103312798 | 1.12 | Up | 4.16E-81 | Peptidoglycan-recognition protein SC1a/b-like |
| LOC663648 | 1.12 | Up | 0.0009606 | Larval/pupal cuticle protein H1C |
| LOC661783 | 1.12 | Up | 8.29E-05 | Solute carrier family 22 member 3-like |
| LOC103314016 | 1.12 | Up | 1.07E-41 | Probable serine/threonine-protein kinase clka |
| LOC661253 | 1.12 | Up | 1.16E-253 | Peptide transporter family 1-like |
| LOC663001 | 1.11 | Up | 0 | Antichymotrypsin-2 |
| LOC100142219 | 1.11 | Up | 2.88E-15 | Kappa-type opioid receptor-like |
| LOC655582 | 1.11 | Up | 1.40E-07 | UPF0605 protein |
| LOC657890 | 1.11 | Up | 2.64E-27 | Calcium release-activated calcium channel protein 1 |
| LOC656094 | 1.11 | Up | 2.96E-18 | Ventral nervous system defective/Vnd |
| LOC664309 | 1.11 | Up | 1.38E-127 | Chaoptic-like protein |
| LOC103313498 | 1.10 | Up | 0 | Crammer |
| LOC103314788 | 1.10 | Up | 0.0001633 | / |
| LOC657728 | 1.10 | Up | 4.27E-13 | Endocuticle structural glycoprotein sgabd-4 |
| LOC663803 | 1.10 | Up | 3.44E-205 | Eukaryotic translation initiation factor 4E-binding protein |
| LOC655201 | 1.10 | Up | 0 | Protein takeout |
| LOC103314892 | 1.10 | Up | 5.56E-27 | / |
| LOC103314100 | 1.09 | Up | 1.68E-18 | Keratin-associated protein 19-2 |
| LOC660944 | 1.09 | Up | 1.34E-208 | Myrosinase 1-like |
| LOC654862 | 1.09 | Up | 5.22E-272 | Proton-coupled amino acid transporter 1 |
| LOC662314 | 1.09 | Up | 0 | Homogentisate 1,2-dioxygenase |
| LOC107397414 | 1.09 | Up | 1.33E-89 | / |
| LOC103312135 | 1.09 | Up | 3.52E-35 | Larval cuticle protein A2B |
| LOC662882 | 1.09 | Up | 8.01E-13 | Peritrophic matrix protein 2-C |
| LOC657573 | 1.09 | Up | 2.10E-06 | Major antigen |
| LOC103312808 | 1.09 | Up | 2.68E-09 | Leucine-rich repeat transmembrane neuronal protein 4 |
| LOC103314145 | 1.09 | Up | 7.63E-47 | / |
| LOC103312897 | 1.09 | Up | 6.98E-06 | IQ domain-containing protein G |
| LOC659787 | 1.09 | Up | 4.52E-106 | Kruppel-homolog 1 |
| LOC657667 | 1.08 | Up | 1.27E-166 | Sodium-independent sulfate anion transporter-like |
| LOC663519 | 1.08 | Up | 5.94E-28 | Cytochrome b561 domain-containing protein 2-like |
| LOC661216 | 1.08 | Up | 2.33E-05 | Glucose dehydrogenase [FAD, quinone] |
| LOC659750 | 1.08 | Up | 2.38E-10 | Nervous wreck-like/nwk |
| LOC662648 | 1.07 | Up | 0.0007834 | Spermine oxidase |
| LOC107399193 | 1.07 | Up | 8.22E-176 | Gametocyte-specific factor 1 homolog |
| LOC657560 | 1.07 | Up | 3.78E-13 | CYP6A14 |
| LOC100142085 | 1.07 | Up | 0 | Peritrophic matrix protein 9 |
| LOC664276 | 1.07 | Up | 5.51E-41 | Protein lethal(2)essential for life |
| LOC658255 | 1.06 | Up | 1.86E-46 | Pyrazinamidase/nicotinamidase |
| LOC659252 | 1.06 | Up | 3.23E-48 | Calpain-A |
| LOC658492 | 1.06 | Up | 1.76E-38 | Eukaryotic translation initiation factor 6-like |
| LOC656573 | 1.06 | Up | 1.39E-34 | Sodium- and chloride-dependent glycine transporter 1 |
| LOC660830 | 1.06 | Up | 1.25E-13 | Glucose dehydrogenase [FAD, quinone] |
| LOC103313592 | 1.06 | Up | 1.13E-12 | / |
| LOC662904 | 1.06 | Up | 0 | Glycine-rich cell wall structural protein |
| LOC103314598 | 1.05 | Up | 3.98E-27 | / |
| LOC103313785 | 1.05 | Up | 1.24E-41 | Natterin-4-like |
| LOC660113 | 1.05 | Up | 3.71E-170 | Fatty acid synthase |
| LOC664099 | 1.05 | Up | 2.06E-193 | Scavenger receptor class B, member 1-like |
| LOC655468 | 1.04 | Up | 7.80E-51 | Cationic amino acid transporter 2 |
| LOC660185 | 1.04 | Up | 1.63E-286 | Phospholipase A12 |
| LOC662942 | 1.04 | Up | 1.49E-265 | / |
| LOC659324 | 1.04 | Up | 3.47E-23 | Peritrophic matrix protein 1-B |
| LOC658415 | 1.04 | Up | 1.31E-14 | Eukaryotic translation initiation factor 6 |
| LOC656587 | 1.04 | Up | 4.24E-25 | Transmembrane channel-like protein 7 |
| LOC103314772 | 1.04 | Up | 4.57E-21 | Cuticlin-1 |
| LOC656084 | 1.04 | Up | 0.0002115 | Cilia-and flagella-associated protein 52 |
| LOC656546 | 1.04 | Up | 3.32E-23 | ATP-dependent DNA helicase PIF1 |
| LOC103314303 | 1.03 | Up | 1.29E-19 | CD63 antigen-like |
| LOC655282 | 1.03 | Up | 1.11E-97 | Spermidine synthase |
| LOC656711 | 1.03 | Up | 3.61E-51 | Serine protease H111 |
| LOC103313660 | 1.03 | Up | 1.29E-14 | Kunitz-type serine protease inhibitor textilinin-3-like |
| LOC103314603 | 1.03 | Up | 1.44E-09 | Putative nuclease HARBI1 |
| LOC657994 | 1.03 | Up | 0 | Serine protease P121 |
| LOC661588 | 1.03 | Up | 4.25E-67 | Arylsulfatase B |
| LOC662360 | 1.02 | Up | 0 | Cuticle protein 21 |
| LOC103314319 | 1.02 | Up | 0.0005984 | / |
| LOC662723 | 1.02 | Up | 9.41E-16 | Camp and camp-inhibited cgmp 3',5'-cyclic phosphodiesterase 10A |
| LOC656559 | 1.02 | Up | 4.39E-182 | Gld |
| LOC107397538 | 1.02 | Up | 3.47E-62 | / |
| LOC664316 | 1.02 | Up | 4.67E-21 | Diuretic hormone receptor |
| LOC661991 | 1.02 | Up | 6.57E-113 | Serine protease H130 |
| LOC657836 | 1.01 | Up | 1.10E-101 | Serine protease H104 |
| LOC659679 | 1.01 | Up | 5.50E-10 | CUGBP Elav-like family member 4 |
| LOC107398192 | 1.01 | Up | 0 | / |
| LOC663683 | 1.01 | Up | 3.29E-154 | / |
| LOC103313730 | 1.01 | Up | 3.04E-269 | Vanin-like protein 1 |
| LOC100142444 | 1.00 | Up | 5.99E-27 | Tachykinin-like receptor at 86C |
| LOC658108 | -1.00 | Down | 3.18E-21 | Cell division cycle protein 20 homolog |
| LOC657403 | -1.00 | Down | 5.48E-13 | Miniature |
| LOC660084 | -1.00 | Down | 7.50E-06 | Protein Wnt-8a/Wnt8/D |
| LOC103313206 | -1.00 | Down | 0.0003248 | / |
| LOC100142197 | -1.00 | Down | 0.0005609 | B6 |
| LOC661334 | -1.00 | Down | 0 | / |
| LOC656613 | -1.01 | Down | 1.68E-20 | DNA polymerase epsilon subunit 2 |
| LOC660274 | -1.01 | Down | 0 | Hexamerin 1B |
| LOC659990 | -1.01 | Down | 1.93E-13 | Facilitated trehalose transporter Tret1 |
| LOC663008 | -1.01 | Down | 2.22E-10 | Soluble guanylate cyclase 89Db |
| LOC664442 | -1.02 | Down | 2.95E-27 | Probable ATP-dependent DNA helicase DDX11 |
| LOC107397402 | -1.02 | Down | 0.0005918 | / |
| LOC660563 | -1.02 | Down | 1.67E-19 | DNA primase large subunit |
| LOC664175 | -1.02 | Down | 5.56E-156 | Netrin receptor UNC5C |
| LOC655424 | -1.02 | Down | 3.15E-50 | Irregular chiasm C-roughest protein |
| LOC661704 | -1.02 | Down | 9.99E-177 | Transmembrane protease serine 9 |
| LOC662397 | -1.02 | Down | 0 | WD40 domain-containing protein |
| LOC100271908 | -1.02 | Down | 4.25E-27 | Vestigial |
| LOC103314786 | -1.03 | Down | 3.69E-08 | Leucine-rich repeat-containing protein egg-6-like |
| LOC660655 | -1.03 | Down | 4.55E-190 | G1/S-specific cyclin-D1 |
| LOC100141771 | -1.03 | Down | 7.26E-160 | Forked end |
| LOC103313423 | -1.03 | Down | 2.35E-23 | Spindle assembly abnormal protein 6 homolog |
| LOC659149 | -1.04 | Down | 0 | Zinc finger SWIM domain-containing protein 8 |
| LOC656598 | -1.04 | Down | 7.79E-08 | Protein Wnt-10a |
| LOC658645 | -1.04 | Down | 0.000263 | Catalase |
| LOC103314438 | -1.04 | Down | 1.16E-25 | / |
| LOC103313062 | -1.04 | Down | 0 | P149/breast cancer type 1 susceptibility protein homolog |
| LOC655734 | -1.04 | Down | 0 | Ransferrin |
| LOC661884 | -1.04 | Down | 0.0003 | Cilia- and flagella-associated protein 47 |
| LOC664605 | -1.04 | Down | 8.43E-107 | Nucleolar protein 4 |
| LOC657218 | -1.04 | Down | 5.45E-90 | Neurogenic protein big brain |
| LOC103314375 | -1.04 | Down | 8.31E-11 | / |
| LOC103312859 | -1.05 | Down | 4.82E-16 | / |
| LOC107397934 | -1.05 | Down | 1.06E-15 | Knockout |
| LOC663752 | -1.05 | Down | 4.33E-158 | Neurotactin |
| LOC661936 | -1.06 | Down | 2.10E-25 | Protein Wnt-7b |
| LOC657169 | -1.06 | Down | 7.08E-80 | Phospholipase B1, membrane-associated |
| LOC100141896 | -1.06 | Down | 0 | Innexin inx3 |
| LOC641551 | -1.06 | Down | 2.54E-88 | Engrailed |
| LOC103313431 | -1.06 | Down | 3.66E-76 | / |
| LOC100141766 | -1.06 | Down | 7.16E-17 | / |
| LOC100141732 | -1.07 | Down | 3.17E-31 | Probable acyl-activating enzyme 21 |
| LOC103313909 | -1.08 | Down | 2.57E-26 | Eater |
| LOC662866 | -1.09 | Down | 4.15E-18 | Venom acid phosphatase Acph-1 |
| LOC103313121 | -1.09 | Down | 8.03E-16 | Serine/threonine-protein kinase fused |
| LOC659613 | -1.09 | Down | 2.91E-15 | Lipase member I |
| LOC659674 | -1.09 | Down | 1.32E-270 | Serine/threonine-protein kinase NLK |
| LOC657914 | -1.09 | Down | 8.17E-228 | Myosin 20 |
| LOC662428 | -1.10 | Down | 0 | Fibroin heavy chain |
| LOC664153 | -1.10 | Down | 0.0004651 | Facilitated trehalose transporter Tret1 |
| LOC663005 | -1.10 | Down | 5.10E-66 | Organic cation transporter protein |
| LOC661027 | -1.10 | Down | 4.18E-194 | Protein mab-21 |
| LOC103314973 | -1.10 | Down | 4.97E-06 | / |
| LOC103313115 | -1.10 | Down | 2.29E-22 | / |
| LOC658883 | -1.10 | Down | 2.71E-199 | / |
| LOC659991 | -1.11 | Down | 9.73E-43 | Claspin |
| LOC103312353 | -1.11 | Down | 7.56E-09 | Integrin beta-1-A-like |
| LOC656698 | -1.11 | Down | 2.40E-18 | Lysosomal alpha-mannosidase-like |
| LOC103314650 | -1.12 | Down | 0.0011505 | / |
| LOC657825 | -1.12 | Down | 1.27E-15 | BG |
| LOC655409 | -1.12 | Down | 1.12E-294 | Actin, muscle |
| LOC103313267 | -1.12 | Down | 5.02E-17 | Larval cuticle protein LCP-30 |
| LOC655955 | -1.12 | Down | 1.14E-140 | Frizzled 4 |
| LOC663899 | -1.12 | Down | 0 | Myosin-9 |
| LOC103315179 | -1.12 | Down | 4.70E-53 | Delta (7)-sterol 5(6)-desaturase erg31 |
| LOC655532 | -1.12 | Down | 1.10E-147 | Impe3-Ecdysone-inducible gene E3 |
| LOC103313774 | -1.13 | Down | 1.30E-08 | Histone-lysine N-methyltransferase eggless |
| LOC103315036 | -1.13 | Down | 1.02E-09 | Centromere protein S |
| LOC658656 | -1.13 | Down | 9.30E-31 | Apterous a |
| LOC660376 | -1.13 | Down | 8.63E-16 | Short-chain dehydrogenase/reductase family 16C member |
| LOC103312846 | -1.13 | Down | 1.27E-19 | / |
| LOC659297 | -1.13 | Down | 0.0001664 | Atonal |
| LOC664392 | -1.13 | Down | 4.06E-33 | Alpha-amylase |
| LOC656688 | -1.14 | Down | 1.88E-31 | Trypsin-3 |
| LOC661412 | -1.14 | Down | 8.42E-24 | Venom acid phosphatase Acph-1 |
| LOC657400 | -1.14 | Down | 6.03E-51 | Trynity |
| LOC100142148 | -1.14 | Down | 5.33E-10 | Chymotrypsin-elastase inhibitor ixodidin-like |
| LOC660913 | -1.14 | Down | 0 | LIX1-like protein |
| LOC103312996 | -1.14 | Down | 4.35E-06 | ADP-ribosylation factor-like protein 2-binding protein |
| LOC662838 | -1.15 | Down | 2.90E-12 | Venom acid phosphatase Acph-1 |
| LOC662206 | -1.15 | Down | 3.28E-30 | Histone H3.3 |
| LOC659304 | -1.15 | Down | 0 | Keratin-associated protein 19-9b |
| LOC656658 | -1.15 | Down | 2.64E-09 | Tubulin epsilon chain |
| LOC656430 | -1.15 | Down | 4.07E-19 | Fork head domain-containing protein FD4 |
| LOC652967 | -1.15 | Down | 0 | Chitinase 10 |
| LOC656355 | -1.16 | Down | 1.57E-27 | Regucalcin |
| LOC656812 | -1.16 | Down | 2.91E-52 | Pair-rule protein odd-paired |
| LOC658877 | -1.16 | Down | 4.20E-28 | Serine protease P162 |
| LOC661379 | -1.16 | Down | 1.14E-36 | Glucose dehydrogenase [FAD, quinone] |
| LOC660577 | -1.16 | Down | 0 | / |
| LOC660796 | -1.16 | Down | 1.88E-06 | Osiris 16 |
| LOC100141699 | -1.16 | Down | 1.54E-112 | / |
| LOC659061 | -1.16 | Down | 2.83E-57 | Lipid storage droplets surface-binding protein 1 |
| LOC660368 | -1.16 | Down | 3.69E-175 | Cat L precursor |
| LOC103315019 | -1.17 | Down | 1.28E-12 | Cuticle protein CP14.6 |
| LOC100142549 | -1.17 | Down | 9.34E-48 | Targeting protein for Xklp2 |
| LOC100142486 | -1.17 | Down | 1.98E-46 | Ugt86Dc |
| LOC659176 | -1.17 | Down | 0 | Endocuticle structural glycoprotein sgabd-5 |
| LOC656349 | -1.17 | Down | 0 | C-type lectin |
| LOC103313102 | -1.17 | Down | 1.86E-21 | Haf |
| LOC107397862 | -1.17 | Down | 2.19E-44 | Interference hedgehog-like Protein |
| LOC107399214 | -1.17 | Down | 2.19E-44 | Exonuclease |
| LOC661758 | -1.18 | Down | 2.03E-83 | Ankyrin repeat and BTB/POZ domain-containing protein 2 |
| LOC661130 | -1.18 | Down | 2.06E-301 | C-type lectin |
| LOC659972 | -1.18 | Down | 3.66E-71 | Protein snail |
| LOC100142350 | -1.18 | Down | 8.58E-05 | / |
| LOC103312478 | -1.18 | Down | 3.49E-31 | Protein APCDD1-like |
| LOC663405 | -1.18 | Down | 1.29E-303 | / |
| LOC661820 | -1.19 | Down | 2.25E-39 | Pannier |
| LOC107397924 | -1.19 | Down | 0.0001193 | Cuticle protein 2-like |
| LOC103312494 | -1.19 | Down | 0 | Tweedleg |
| LOC103315077 | -1.19 | Down | 3.17E-06 | / |
| LOC100141918 | -1.19 | Down | 1.84E-11 | Somatostatin receptor type 4 |
| LOC664440 | -1.20 | Down | 1.07E-35 | GILT-like protein F37H8.5 |
| LOC103313224 | -1.20 | Down | 1.02E-151 | Extensin-like |
| LOC662243 | -1.20 | Down | 1.91E-20 | Werner Syndrome-like exonuclease |
| LOC656569 | -1.20 | Down | 3.50E-36 | Kinesin-like protein KIF11-B |
| LOC660815 | -1.21 | Down | 2.54E-251 | Tyrosine-protein kinase-like otk |
| LOC103314387 | -1.21 | Down | 2.41E-59 | Kinetochore protein ndc80 |
| LOC660862 | -1.21 | Down | 2.77E-25 | Ras-related and estrogen-regulated growth inhibitor |
| LOC656306 | -1.22 | Down | 3.25E-57 | CYP4BN5 |
| LOC656334 | -1.22 | Down | 9.15E-69 | Kelch-like protein 21 |
| LOC662692 | -1.22 | Down | 1.86E-100 | Probable RNA-directed DNA polymerase from transposon X-element |
| LOC107397457 | -1.22 | Down | 7.91E-09 | Peritrophin-1-like |
| LOC641604 | -1.22 | Down | 2.50E-05 | Orthodenticle-2 |
| LOC103314645 | -1.23 | Down | 2.80E-178 | Gustatory receptor candidate 59 |
| LOC658369 | -1.23 | Down | 6.32E-84 | / |
| LOC664207 | -1.23 | Down | 2.09E-248 | Protocadherin Fat 3-like isoform X1 |
| LOC103314889 | -1.24 | Down | 6.67E-12 | / |
| LOC662799 | -1.24 | Down | 0 | Cuticular protein |
| LOC657597 | -1.24 | Down | 2.67E-73 | Circadian clock-controlled protein |
| LOC655331 | -1.24 | Down | 5.47E-242 | GSTe7 |
| LOC660248 | -1.25 | Down | 1.94E-147 | / |
| LOC103313235 | -1.25 | Down | 2.95E-24 | / |
| LOC660572 | -1.26 | Down | 1.48E-135 | Fatty acyl-coa reductase 1 |
| LOC100142410 | -1.26 | Down | 1.35E-28 | Insulin-like growth factor-binding protein complex acid labile subunit |
| LOC107398449 | -1.26 | Down | 1.41E-13 | Transmembrane protein 203 |
| LOC107397404 | -1.26 | Down | 9.66E-27 | Luciferin 4-monooxygenase-like |
| LOC655811 | -1.26 | Down | 1.64E-41 | Brachyurin |
| LOC103312140 | -1.26 | Down | 1.14E-81 | Larval cuticle protein 8 |
| LOC103312748 | -1.27 | Down | 5.86E-105 | Peritrophin-1-like |
| LOC103313179 | -1.27 | Down | 3.71E-40 | G2/mitotic-specific cyclin-B |
| LOC656829 | -1.27 | Down | 6.07E-10 | Transmembrane protein 80 |
| LOC655301 | -1.27 | Down | 1.70E-58 | Condensin complex subunit 2 |
| LOC662271 | -1.27 | Down | 6.66E-299 | Mite allergen Der p 7-like |
| LOC664235 | -1.27 | Down | 9.76E-72 | Serine protease inhibitor dipetalogastin |
| LOC658287 | -1.27 | Down | 6.72E-294 | Farnesol dehydrogenase-like |
| LOC100142126 | -1.28 | Down | 0.0006722 | NADH dehydrogenase (ubiquinone) MWFE subunit |
| LOC659285 | -1.28 | Down | 1.23E-48 | / |
| LOC662197 | -1.28 | Down | 9.74E-90 | Alpha-N-acetylgalactosaminidase |
| LOC103312472 | -1.29 | Down | 1.59E-34 | / |
| LOC660272 | -1.29 | Down | 1.07E-44 | Trans-1,2-dihydrobenzene-1,2-diol dehydrogenase |
| LOC658740 | -1.29 | Down | 4.06E-35 | / |
| LOC641600 | -1.29 | Down | 2.78E-87 | Scr |
| LOC100141826 | -1.30 | Down | 7.83E-13 | Odorant receptor 22c |
| LOC657243 | -1.30 | Down | 0 | Innexin inx2 |
| LOC655120 | -1.30 | Down | 2.34E-05 | Elastin |
| LOC663117 | -1.30 | Down | 7.73E-28 | Cat B1 |
| LOC655833 | -1.31 | Down | 1.31E-194 | Mex-3 protein |
| LOC658140 | -1.31 | Down | 0 | High mobility group protein I-like |
| LOC662160 | -1.31 | Down | 9.24E-22 | TIMELESS-interacting protein |
| LOC664509 | -1.31 | Down | 0 | Enolase-phosphatase E1 |
| LOC663954 | -1.31 | Down | 0 | Alpha amylase |
| LOC660251 | -1.31 | Down | 3.53E-21 | / |
| LOC656390 | -1.31 | Down | 2.43E-45 | / |
| LOC655012 | -1.32 | Down | 9.86E-71 | Mushroom body large-type Kenyon cell-specific protein 1 |
| LOC663553 | -1.32 | Down | 1.62E-40 | Pregnancy zone protein |
| LOC659525 | -1.32 | Down | 5.41E-84 | Arylsulfatase B |
| LOC659841 | -1.33 | Down | 4.79E-14 | LIM/homeobox protein Awh |
| LOC655415 | -1.33 | Down | 3.03E-92 | GSTe8 |
| LOC662771 | -1.35 | Down | 1.67E-16 | Endocuticle structural glycoprotein sgabd-2 |
| LOC100142362 | -1.35 | Down | 3.54E-26 | / |
| LOC103312615 | -1.36 | Down | 1.41E-07 | Zonadhesin-like |
| LOC655752 | -1.36 | Down | 9.57E-12 | Serine protease P38 |
| LOC664065 | -1.37 | Down | 5.93E-175 | / |
| LOC103313871 | -1.37 | Down | 6.01E-217 | Mucin-12-like |
| LOC100141678 | -1.37 | Down | 7.30E-71 | / |
| LOC107399163 | -1.37 | Down | 0.0010138 | Rrna biogenesis protein rrp36-like |
| LOC658383 | -1.37 | Down | 3.89E-184 | Metabotropic glutamate receptor 3 |
| LOC100142248 | -1.37 | Down | 0 | Macrophage migration inhibitory factor homolog |
| LOC658217 | -1.38 | Down | 8.17E-06 | Protein phosphatase 1 regulatory subunit 42 |
| LOC659104 | -1.38 | Down | 3.17E-32 | Vanin-like protein 2 |
| LOC100142166 | -1.38 | Down | 3.94E-90 | Sortilin-related receptor |
| LOC103313083 | -1.38 | Down | 3.57E-05 | / |
| LOC100142269 | -1.39 | Down | 7.87E-37 | Zinc finger protein 358 |
| LOC660573 | -1.40 | Down | 8.03E-89 | C-type lectin |
| LOC100141645 | -1.40 | Down | 1.03E-09 | / |
| LOC641598 | -1.41 | Down | 5.97E-60 | ABC transmembrane transporter white |
| LOC656466 | -1.41 | Down | 7.38E-28 | Luciferin 4-monooxygenase |
| LOC662133 | -1.42 | Down | 4.09E-06 | G2/mitotic-specific cyclin-A |
| LOC658886 | -1.42 | Down | 1.80E-08 | XDH |
| LOC663744 | -1.42 | Down | 1.71E-15 | / |
| LOC103314924 | -1.42 | Down | 4.10E-275 | RNA polymerase II degradation factor 1 |
| LOC103313983 | -1.43 | Down | 3.00E-30 | Dumpy |
| LOC661734 | -1.44 | Down | 4.99E-93 | Putative fatty acyl-coa reductase |
| LOC660033 | -1.45 | Down | 0 | Pleiotrophin |
| LOC656776 | -1.45 | Down | 0 | Putative fatty acyl-coa reductase CG5065 |
| LOC660001 | -1.45 | Down | 6.54E-05 | Alkaline phosphatase |
| LOC655214 | -1.46 | Down | 3.61E-168 | Facilitated trehalose transporter Tret1 |
| LOC664389 | -1.46 | Down | 1.21E-28 | Alpha-amylase-like |
| LOC107399018 | -1.46 | Down | 4.44E-103 | / |
| LOC662392 | -1.47 | Down | 0.0007459 | Histone H2A |
| LOC662504 | -1.47 | Down | 6.36E-08 | Mucin 68e |
| LOC103312650 | -1.47 | Down | 0.0008053 | Transient receptor potential channel pyrexia |
| LOC661967 | -1.48 | Down | 7.35E-79 | Ugt2B20 |
| LOC100141947 | -1.48 | Down | 4.02E-52 | Attacin 2 |
| LOC103313683 | -1.48 | Down | 0.0006198 | / |
| LOC657761 | -1.50 | Down | 0 | Acyl-coa synthetase short-chain family member 3, mitochondrial |
| LOC100141646 | -1.51 | Down | 1.46E-64 | Cecropin 2 |
| LOC659878 | -1.51 | Down | 1.31E-71 | CYP4Q1 |
| LOC657808 | -1.53 | Down | 0 | Endocuticle structural glycoprotein sgabd-8 |
| LOC103315141 | -1.53 | Down | 5.03E-05 | / |
| LOC664041 | -1.54 | Down | 0 | Alpha-amylase-like |
| LOC660225 | -1.54 | Down | 5.31E-92 | Alpha-L-fucosidase-like |
| LOC659390 | -1.55 | Down | 4.76E-130 | Cgmp-dependent 3',5'-cyclic phosphodiesterase |
| LOC107398098 | -1.55 | Down | 0.0003478 | Cilia- and flagella-associated protein 61 |
| LOC107398667 | -1.55 | Down | 0.0005917 | Odorant receptor 59c |
| LOC662899 | -1.56 | Down | 0 | Fatty acid synthase |
| LOC658244 | -1.56 | Down | 0 | / |
| LOC103315057 | -1.56 | Down | 2.18E-05 | Antimeros |
| LOC103313174 | -1.57 | Down | 3.34E-70 | / |
| LOC658755 | -1.57 | Down | 3.74E-09 | Vitellogenin |
| LOC661084 | -1.58 | Down | 1.70E-87 | Protein mab-21 |
| LOC103313138 | -1.59 | Down | 1.32E-76 | Kinesin-II 85 kda subunit-like |
| LOC659193 | -1.60 | Down | 0 | Broad-complex |
| LOC103312570 | -1.62 | Down | 0.0002563 | Thyrotropin-releasing hormone receptor |
| LOC103312725 | -1.63 | Down | 3.19E-11 | ML |
| LOC655161 | -1.64 | Down | 6.13E-06 | Fibronectin type 3 and ankyrin repeat domains 1 protein |
| LOC662448 | -1.64 | Down | 0 | Somatomedin-B and thrombospondin type-1 domain-containing protein |
| LOC659803 | -1.65 | Down | 4.49E-29 | Protein NDNF |
| LOC664022 | -1.65 | Down | 0 | Alpha-amylase |
| LOC103313521 | -1.65 | Down | 2.03E-15 | / |
| LOC659012 | -1.66 | Down | 0 | Flexible cuticle protein 12 |
| LOC655764 | -1.66 | Down | 0 | Peritrophic matrix protein 5-B |
| LOC662371 | -1.67 | Down | 1.09E-34 | Glycine N-methyltransferase |
| LOC107399224 | -1.67 | Down | 1.40E-17 | Trypsin-1 |
| LOC107397724 | -1.68 | Down | 2.10E-08 | / |
| LOC664318 | -1.69 | Down | 1.03E-23 | Histone H3 |
| LOC658508 | -1.69 | Down | 5.83E-181 | Sarcoplasmic calcium-binding protein |
| LOC100142481 | -1.70 | Down | 4.05E-38 | Attacin 1 |
| LOC660918 | -1.70 | Down | 2.05E-32 | Trypsin-1 |
| LOC103314503 | -1.71 | Down | 1.57E-08 | Choline transporter-like protein 1 |
| LOC100142057 | -1.73 | Down | 0 | Stress response protein NST1 |
| LOC662691 | -1.73 | Down | 2.16E-42 | / |
| LOC103314072 | -1.73 | Down | 0 | Peritrophic matrix protein 5-B |
| LOC658671 | -1.73 | Down | 1.49E-211 | Farnesol dehydrogenase |
| LOC662735 | -1.74 | Down | 3.27E-90 | Endocuticle structural glycoprotein sgabd-2 |
| LOC664046 | -1.75 | Down | 3.53E-83 | / |
| LOC661631 | -1.75 | Down | 8.99E-81 | Lipase 1 |
| LOC107398113 | -1.76 | Down | 1.96E-13 | / |
| LOC103314751 | -1.76 | Down | 1.70E-34 | Coleoptericin-like |
| LOC660458 | -1.77 | Down | 2.70E-52 | Microtubule-associated proteins 1A/1B light chain 3C |
| LOC659587 | -1.78 | Down | 0 | Arylsulfatase B |
| LOC103312546 | -1.80 | Down | 4.30E-68 | Brain tumor protein |
| LOC103314652 | -1.80 | Down | 7.18E-17 | / |
| LOC103314024 | -1.81 | Down | 5.57E-43 | Enhancer of split malpha protein |
| LOC100142559 | -1.81 | Down | 2.88E-166 | Carboxypeptidase inhibitor precursor |
| LOC659121 | -1.82 | Down | 2.14E-132 | Lipase 1 like |
| LOC660559 | -1.82 | Down | 0 | Pollen-specific leucine-rich repeat extensin-like protein 1 |
| LOC661012 | -1.82 | Down | 9.09E-11 | Brachyurin-like |
| LOC100141679 | -1.83 | Down | 1.90E-26 | Bromodomain-containing protein DDB_G0280777 |
| LOC100142396 | -1.83 | Down | 8.15E-20 | Nicotinate phosphoribosyltransferase |
| LOC103313036 | -1.84 | Down | 0.0010579 | / |
| LOC103314753 | -1.84 | Down | 6.55E-41 | Coleoptericin |
| LOC103312173 | -1.84 | Down | 1.69E-05 | Probable RNA-binding protein 46 |
| LOC658941 | -1.85 | Down | 1.89E-27 | Flexible cuticle protein 12 |
| LOC100141789 | -1.85 | Down | 2.53E-74 | Serine protease P40 |
| LOC660027 | -1.86 | Down | 4.38E-13 | Cuticular protein analogous to peritrophins 3-A2 |
| LOC658103 | -1.88 | Down | 1.64E-48 | Z9desA |
| LOC103314783 | -1.88 | Down | 5.25E-12 | E3 ubiquitin-protein ligase MARCH3 |
| LOC100142608 | -1.89 | Down | 0.0006585 | / |
| LOC107398700 | -1.89 | Down | 0.0006525 | Scraps |
| LOC103313098 | -1.92 | Down | 2.69E-08 | / |
| LOC103312222 | -1.93 | Down | 2.59E-09 | Peritrophin-1-like |
| LOC103313418 | -1.94 | Down | 2.65E-25 | Zinc finger protein GLIS1 |
| LOC656885 | -1.95 | Down | 3.25E-10 | Glucose dehydrogenase [FAD, quinone] |
| LOC103313377 | -1.96 | Down | 0 | SGNH/GDSL hydrolase family protein |
| LOC107399077 | -1.96 | Down | 0.0011143 | / |
| LOC103314047 | -1.97 | Down | 7.97E-199 | Epidermal growth factor-like protein 8 |
| LOC103312352 | -1.97 | Down | 5.69E-100 | Integrin beta-PS-like |
| LOC107398282 | -1.98 | Down | 2.23E-205 | Cuticle protein 16.5-like |
| LOC662300 | -1.99 | Down | 3.03E-22 | CYP351A3 |
| LOC656629 | -2.00 | Down | 8.51E-98 | Defensin 2 |
| LOC103314254 | -2.01 | Down | 1.92E-14 | Odorant receptor 76 |
| LOC659737 | -2.04 | Down | 5.35E-05 | Neuropeptide Y receptor |
| LOC658305 | -2.09 | Down | 9.43E-17 | Muts protein homolog 5 |
| LOC664063 | -2.10 | Down | 0 | Pathogenesis-related protein 5 |
| LOC658009 | -2.11 | Down | 5.06E-60 | Histone H2B |
| LOC657457 | -2.11 | Down | 0 | Pathogenesis-related protein 5 |
| LOC661718 | -2.14 | Down | 6.36E-24 | Lipase 3 like |
| LOC661799 | -2.15 | Down | 9.46E-10 | CSP8 |
| LOC103313450 | -2.18 | Down | 8.21E-71 | / |
| LOC656563 | -2.19 | Down | 4.24E-39 | Histone H2B |
| LOC659859 | -2.20 | Down | 0 | Hornerin |
| LOC103312508 | -2.20 | Down | 5.29E-29 | Endo-alpha-mannosidase |
| LOC103313826 | -2.22 | Down | 8.77E-05 | FMO 2 |
| LOC103313095 | -2.24 | Down | 5.88E-277 | / |
| LOC664598 | -2.25 | Down | 0 | OBPC01 |
| LOC103315235 | -2.25 | Down | 9.69E-207 | Probable chitinase 3 |
| LOC103314122 | -2.28 | Down | 0 | Prisilkin-39 |
| LOC664385 | -2.28 | Down | 1.65E-211 | Alpha-amylase |
| LOC103313668 | -2.32 | Down | 7.34E-08 | Poly [ADP-ribose] polymerase 11 |
| LOC103313231 | -2.32 | Down | 0.0001412 | Neprilysin-21-like |
| LOC103313522 | -2.33 | Down | 0 | Histidine-rich glycoprotein |
| LOC641601 | -2.35 | Down | 0 | Chitinase 5 |
| LOC660846 | -2.44 | Down | 7.10E-103 | Ugt2C1 |
| LOC103313050 | -2.49 | Down | 5.70E-28 | Trans-1,2-dihydrobenzene-1,2-diol dehydrogenase-like |
| LOC662337 | -2.49 | Down | 6.97E-127 | CYP4C1 |
| LOC664576 | -2.59 | Down | 1.54E-07 | Myrosinase 1 |
| LOC103315062 | -2.59 | Down | 0.0005923 | / |
| LOC657063 | -2.63 | Down | 4.46E-65 | / |
| LOC103313448 | -2.72 | Down | 5.59E-14 | / |
| LOC656983 | -2.76 | Down | 0 | / |
| LOC100142033 | -2.76 | Down | 4.01E-109 | / |
| LOC107398495 | -2.77 | Down | 7.86E-25 | GSTe4 like |
| LOC100142120 | -2.82 | Down | 5.09E-15 | Glycine-rich protein DOT1 |
| LOC103314863 | -2.85 | Down | 6.50E-20 | / |
| LOC664475 | -2.87 | Down | 5.91E-05 | CYP6A20 |
| LOC103312877 | -2.91 | Down | 0.0008532 | Fs(1)N - female sterile (1) Nasrat |
| LOC103314039 | -2.93 | Down | 1.01E-07 | Protein vav-1 |
| LOC661966 | -2.97 | Down | 2.16E-296 | Lipase 4 |
| LOC107398031 | -3.07 | Down | 9.72E-32 | Lymphocyte antigen 6D |
| LOC661930 | -3.09 | Down | 5.08E-05 | CYP349A1 |
| LOC662384 | -3.13 | Down | 0 | Defensin 1 |
| LOC658418 | -3.27 | Down | 1.02E-100 | Zinc finger protein 512B |
| LOC103312521 | -3.33 | Down | 1.94E-40 | / |
| LOC103314862 | -3.46 | Down | 3.89E-14 | Transcription factor mafa-like |
| LOC107398114 | -3.52 | Down | 7.28E-06 | / |
| LOC103314414 | -3.81 | Down | 0.0003061 | / |
| LOC661455 | -3.84 | Down | 4.64E-34 | Venom acid phosphatase Acph-1 |
| LOC103313811 | -4.49 | Down | 0.0006924 | Extensin-like |
| LOC103313213 | -4.62 | Down | 6.06E-35 | / |
| LOC661496 | -4.70 | Down | 4.13E-43 | Venom acid phosphatase Acph-1 |
| LOC107398314 | -5.16 | Down | 1.97E-18 | / |
| LOC107398308 | -6.25 | Down | 1.54E-10 | / |
| LOC657309 | -6.30 | Down | 7.89E-11 | Proteasome subunit beta type-6 |
| LOC107398416 | -6.32 | Down | 5.63E-11 | Odorant receptor 45b |
| LOC107397501 | -7.61 | Down | 6.99E-23 | / |
| LOC107397972 | -8.32 | Down | 4.24E-34 | / |
| LOC107398257 | -8.32 | Down | 4.24E-34 | / |
| LOC655735 | -8.58 | Down | 1.37E-39 | Exonuclease |
| LOC107398725 | -9.43 | Down | 8.21E-46 | / |
| LOC107397403 | -9.92 | Down | 4.62E-82 | / |
| LOC107398411 | -10.48 | Down | 2.09E-34 | / |

Note: C, control; 5% *A. vulgaris* treatment; An *P*-value < 0.05 and the absolute value of the log_2_Ratio ≥ 1 were used as the threshold to judge the significance of gene expression difference. The protein name abbreviation was shown in tables 2-4.
