# Supplementary material for: Insecticidal Activity of Artemisia vulgaris Essential Oil and Transcriptome Analysis of Tribolium castaneum in Response to Oil Exposure
Source: Front Genet. 2020 Jun 25;11:589. doi: 10.3389/fgene.2020.00589 (PMC7330086; doi:10.3389/fgene.2020.00589)
Supplement: TABLE S4 — Differentially expressed genes (DEGs) involved in response to stimuli. [file Table_4.docx]

**Supplementary Table S4** DEGs involved in response to stimulus

| Gene ID | Log_2_Ratio (T/C) | Regulation (T/C) | *P*-value | Protein |
| --- | --- | --- | --- | --- |
| LOC664422 | 2.50 | Up | 0 | Gadd45 |
| LOC662363 | 2.05 | Up | 0 | ADAMTS7 |
| LOC655587 | 2.02 | Up | 2.13E-51 | Peroxidase |
| LOC100141722 | 1.58 | Up | 7.54E-05 | Octopamine receptor 1 |
| LOC660084 | -1.00 | Down | 7.50E-06 | Wnt 8 |
| LOC664175 | -1.02 | Down | 5.56E-156 | Netrin receptor UNC5C |
| LOC658645 | -1.04 | Down | 0 | Catalase |
| LOC103313102 | -1.17 | Down | 1.86E-21 | Haf |
| LOC662160 | -1.31 | Down | 9.24E-22 | TIMELESS-interacting protein |
| LOC103314751 | -1.76 | Down | 1.70E-34 | Coleoptericin-like |
| LOC103314024 | -1.81 | Down | 5.57E-43 | Enhancer of split malpha protein |
| LOC103314753 | -1.84 | Down | 6.55E-41 | Coleoptericin |
| LOC656629 | -2.00 | Down | 8.51E-98 | Defensin 2 |
| LOC659737 | -2.04 | Down | 5.35E-05 | Neuropeptide Y receptor |
| LOC662384 | -3.13 | Down | 0 | Defensin 1 |

Note: C, Control; T, 5% *A. vulgaris* treatment; Gadd45, Growth arrest and DNA damage-indueible genes; ADAMTS7, A disintegrin and metalloproteinase with thrombospondin motifs 7; Haf, Hattifattener.
