# Supplementary material for: Insecticidal Activity of Artemisia vulgaris Essential Oil and Transcriptome Analysis of Tribolium castaneum in Response to Oil Exposure
Source: Front Genet. 2020 Jun 25;11:589. doi: 10.3389/fgene.2020.00589 (PMC7330086; doi:10.3389/fgene.2020.00589)
Supplement: TABLE S5 — Differentially expressed genes (DEGs) related to antigen processing and presentation. [file Table_5.docx]

**Supplementary Table S5** DEGs related to antigen processing and presentation

| Gene ID | Log_2_Ratio (T/C) | Regulation (T/C) | *P*-value | Protein | Phenotype after RNAi of DEGs in *T. castaneum* ^a^ |
| --- | --- | --- | --- | --- | --- |
| LOC100142317 | 3.08 | Up | 0 | HSP68a | / |
| LOC658343 | 2.92 | Up | 1.06E-24 | CP 1 | / |
| LOC100142517 | 2.87 | Up | 0 | HSP68b | / |
| LOC660669 | 2.62 | Up | 8.30E-75 | Cat L precursor | / |
| LOC659367 | 2.08 | Up | 1.84E-77 | Cat L precursor | / |
| LOC657117 | 1.81 | Up | 1.26E-06 | Cat B1 like | / |
| LOC107398158 | 1.79 | Up | 7.41E-05 | Cat L1 like | / |
| LOC663293 | 1.48 | Up | 0 | HSP70a | / |
| LOC656451 | 1.39 | Up | 2.10E-15 | HSP70b | / |
| LOC660551 | 1.26 | Up | 1.04E-307 | Cat L like | / |
| LOC103313498 | 1.10 | Up | 0 | Crammer | / |
| LOC660368 | -1.16 | Down | 3.69E-175 | Cat L precursor | 100% ^b^, 40% vitellogenic egg chamber not present ^d^ |
| LOC664440 | -1.20 | Down | 1.07E-35 | GILT-like protein F37H8.5 | 80% embryo/egg not developed/not fertilized ^c^ |
| LOC663117 | -1.30 | Down | 7.73E-28 | Cat B1 like | / |

Note: C, Control; T, 5% *A. vulgaris* treatment; HSP, Heat shock protein; CP, Cysteine proteinase; Cat, Cathepsin; ^a^ Metamorphosis and survival after RNAi were analyzed (http://ibeetle-base.uni-goettingen.de); ^b^ Lethalities 11 days after larval injection (includes death as larva, prepupa, pupa); ^c^ 14 days after female pupal injection; ^d^ 13 days after female pupal injection.
